# A Review on Research and Evaluation Methods for Investigating Self-Transcendence

**DOI:** 10.3389/fpsyg.2020.547687

**Published:** 2020-11-16

**Authors:** Alexandra Kitson, Alice Chirico, Andrea Gaggioli, Bernhard E. Riecke

**Affiliations:** ^1^School of Interactive Arts and Technology, Simon Fraser University, Burnaby, BC, Canada; ^2^Department of Psychology, Catholic University of the Sacred Heart, Milan, Italy; ^3^ATN-P Lab, Istituto Auxologico Italiano, Milan, Italy

**Keywords:** self-transcendence, review, research methods, mindfulness, flow, peak experiences, neurophenomenolgy, emotional experiences

## Abstract

Self-transcendence has been characterized as a decrease in self-saliency (ego disillusionment) and increased connection, and has been growing in research interest in the past decade. Several measures have been developed and published with some degree of psychometric validity and reliability. However, to date, there has been no review systematically describing, contrasting, and evaluating the different methodological approaches toward measuring self-transcendence including questionnaires, neurological and physiological measures, and qualitative methods. To address this gap, we conducted a review to describe existing methods of measuring self-transcendence, evaluate the strengths and weaknesses of these methods, and discuss research avenues to advance assessment of self-transcendence, including recommendations for suitability of methods given research contexts.

## 1. Introduction

Self-transcendence is often defined as decreased self-saliency and increased connection to others and the environment (Yaden et al., 2017a). However, if we look across different disciplines, we find the focus and conceptualization of self-transcendence varies. For example, psychologists often view self-transcendence as involving an elevation or a separation of self from the environment, whereas nursing regards self-transcendence as awareness of one's wholeness in person-environment connections when fragmentation threatens one's well-being (Smith and Liehr, 2014). Self-transcendence seems to be emerging in several disciplines including nursing theory, developmental psychology, gerontology, personality theory and psychiatric genetics, positive psychology, and others (Garcia-Romeu, 2010). Self-transcendence can be regarded as a psychological state, personality trait, developmental process, value orientation, motivation, and worldview (Wong, 2016). Self-transcendence experience is also considered multifaceted in itself, composed of mindfulness, flow, self-transcendent emotions, awe, peak experiences, and mystical experiences (Yaden et al., 2017a). Therefore, depending on the discipline and focus, one will find very different approaches to investigate the phenomenon of self-transcendence (Garcia-Romeu, 2010). Nonetheless, here we will review and describe the research and evaluation methods across different disciplines for investigating self-transcendence by critically discussing, comparing and evaluating them.

Our goals for this paper are to (1) describe existing methods of measuring self-transcendence; (2) evaluate the strengths and weaknesses of these methods; (3) discuss research avenues to advance assessment of self-transcendence, including recommendations for suitability of methods given research contexts.

## 2. Theories of Self-Transcendence

There exist several theories on self-transcendence, which I divided into three categories: conceptual, phenomenological, and physiological. Each offer a different perspective into understanding self-transcendence since the construct itself is not widely agreed upon.

### 2.1. Conceptual

Frankl (1966) is perhaps one of the first researchers to go against Freudian models of pleasure seeking and equilibrium as our sole drivers and goals, and toward a model of self-transcendence. Here, Frankl's self-transcendence construct emphasizes focusing on serving others and not on fulfilling one's own potential through constant self-referral. A more contemporary model of self-transcendence uses Frankl's construct and describes it on three levels (Wong, 2016):

1. Seeking ultimate meaning —seeking ultimate ideals of goodness, truth, and beauty;
2. Seeking situational meaning —mindful of present moment with openness, curiosity, and compassion;
3. Seeking one's calling —pursue higher purpose for the greater good.

Another key theory that is relevant to today's conceptualization of self-transcendence is Maslow (1943)'s hierarchy of needs (from bottom to top): physiological (survival) through basic life necessities; safety and security through law and order; belongingness and love through group affiliation; esteem through recognition and achievement; self-actualization through fulfillment of personal potential. Theorists postulate there is a sixth tier need—self-transcendence (Venter, 2017). Maslow wanted to show there is a false dichotomy of thinking of the world as only the self and the environment. One who has self-transcendended no longer relies on others' opinions and is free from culture and social environment. Self-transcendence is being no longer grounded or anchored in one's own culture alone; not exclusively defined by their immediate environment or group. Through self-transcending culture, one can better identify with others. Stellar et al. (2017) and Haidt and Morris (2009) also supported the idea that self-transcendent emotions —compassion, awe, gratitude, appreciation, inspiration, admiration, elevation, and love —are key to positive social functioning and connecting to others. For example, compassion helps to support those in need; gratitude builds commitment to others in need; awe reduces self-importance.

As a way to conceptualize self-transcendence, some have suggested self-transcendence is a measurable personality trait that captures the degree to which an individual feels a part of nature and the universe at large (Cloninger et al., 1993a). Four traits predictive of self-transcendence were neuroticism (negatively correlated), openness to experience (positive), agreeableness (positive), and conscientiousness (positive); another predictor variable was meditation practice (positive) (Levenson et al., 2005). Others have derived components of self-transcendence from the aging process and development across the lifespan, otherwise known as *gerotranscendence* (Tornstam, 1994). Yaden et al. (2017a) posit the following constructs for self-transcendence: mindfulness, flow, positive emotions (elevation, compassion, admiration, gratitude, love, awe), peak experiences (e.g., comic consciousness, merging with the universe), mystical experiences (e.g., psychedelic), pathological experiences (e.g., schizophrenia, depersonalization disorder). The authors suggest that self-transcendent experiences contain two sub components: reduced self-saliency (annihilation component) and increased connectedness (relational component). Furthermore, blurring the lines between social and spatial (culture and environment) may be a way to increase perceived social connection and, thus, increase well-being. From Yaden et al. (2017a)'s perspective, self-transcendent experiences do not seem to serve any individualistic evolutionary purpose, but might be seen as a way to reinforce cohesive social groups. Additionally, they are also not well-understood in terms of efficacy, contraindications, and implications for therapeutic purpose.

A highly related construct of self-transcendence is meditation, especially those practices that follow the dissolution of self, time, and external reality. As described in Yaden et al. (2017a) and detailed in Vago and Silbersweig (2012), cultivating a state of mindfulness leads to the development of self-other relations that transcends selfish needs, described in the literature as “decentering.” In advanced mindfulness practitioners, this self-other distinction is completely dissolved (Vago and Zeidan, 2016). Dorjee (2016) describes a contemplative practice framework that outlines the progression in shifts of self and reality as states associated with increasing gradients of dereification. Here, the decentering aspect of mindfulness-based practices has been linked to the initial stages of this progression. Schoenberg and Vago (2019) outline a multi-dimensional model of meditation that progresses in five stages: (1) relaxation practices characterized by neuro-visceral processes; (2) concentration practices characterized by focused and diffuse attention; (3) insight practices characterized by ordinary insight that all objects are illusory or constructs of the mind; (4) non-dual practices characterized by dissolution of the self and everything; (5) cumulative practices characterized by unifying compassion and unconditional love as outcomes of the previous four practices. Self-transcendence in particular seems to relate to the latter stages of this model where the ego is dissolved and meditation practitioners experience a feeling of unity. This concept of different developmental-stages in meditation is echoed in Piron (2001)'s paper on meditative depth, which defines meditation not only in type but also in terms of level felt during different times, i.e., a person can experience different degrees of self-transcendence at different times in their life. Piron (2001) refers to Engel (1997)'s Meditation Development Index (MDI) measure that is based on Engel (1997)'s Zen developmental theory of meditation comprised of eight stages: (1) premeditative: meditation as tranquilizer; (2) searching: uncertainty and disorientation; (3) effort: struggle, fight; (4) level of work: calm, regular exercises; (5) support: pleasantness and motivation to continue; (6) being uplifted; (7) resolution; and (8) afterwards. Piron (2001) then furthered MDI by constructing both the Meditation Depth Index (MEDI) and Meditation Depth Questionnaire (MEDEQ) to measure the greatest depth of meditation and meditation itself in a more differentiated way than the index, respectfully.

Millière et al. (2018) approaches self-transcendence as a multidimensional model of altered self-consciousness. They describe self-consciousness as dissolving the sense of self through meditation and the phenomenon occurring from drug-induced ego-dissolution. Here, self-consciousness is organized into two categories of narrative and embodied selfhood. These two aspects are then plotted against six dimensions: (1) a sense of body ownership, (2) awareness of bodily sensations, (3) awareness of spatial self-location, (4) rich phenomenology, (5) access to semantic autobiographical information, and (6) self-related thoughts. A total loss of self is then the absence of these dimensions for both the narrative and embodied self. The authors have emphasized that self-consciousness is not a simple nor uni-dimensional construct, showing that different forms of meditation and psychedelic states are mapped differently to their multidimensional model.

**Figure 1:**
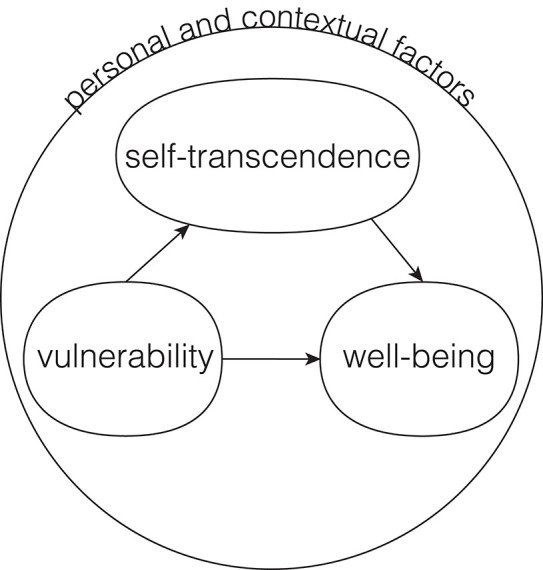
Model of Self-transcendence in the context of nursing described in Reed (2013).

**Figure 2:**
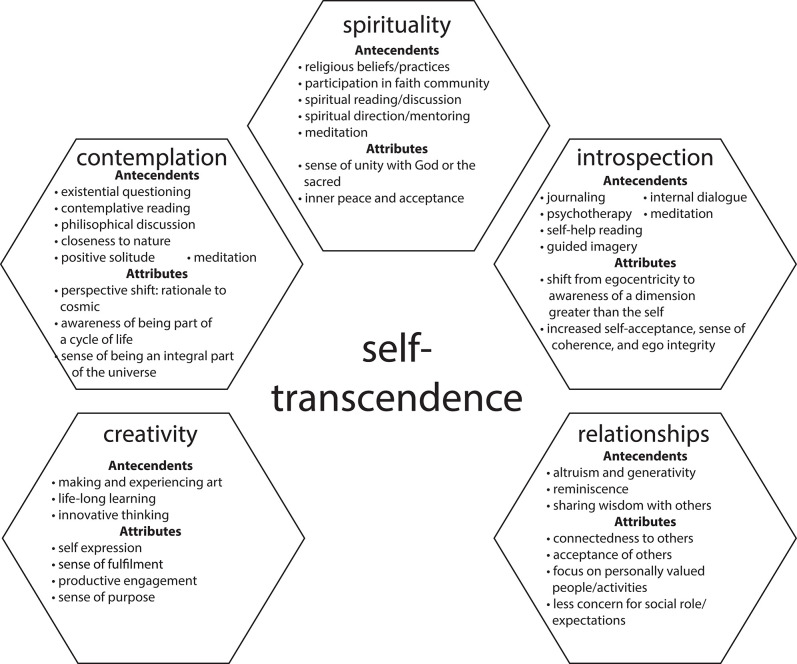
Conceptual model of self-transcendence described in McCarthy et al. (2018). This model was originally used for the Psychoeducational Approach to Transcendence and Health (PATH) program on self-transcendence and well-being in community-dwelling older adults.

Nursing's conceptual idea of self-transcendence is “the capacity to expand self-boundaries in a variety of ways” (Reed, 2013). In forming this concept of self-transcendence, nurses drew from Neo-Piagetian theories about development in adulthood and later life. The model itself is composed of four basic sets of relationships (Figure 1). First, increased levels of vulnerability, e.g., health event, influence increased levels of self-transcendence. Reed describes vulnerability here as the awareness of one's own mortality, and that self-transcendence arises naturally from this awareness that is often triggered by life crises. Second, self-transcendence relates positively to a sense of well-being and morale but relates negatively to the level of depression. Third, self-transcendence mediates the effects of vulnerability on well-being. Fourth, personal and contextual factors play a role in all three variables, e.g., age, gender, cognitive ability, health status, past significant life events, personal beliefs, family support, and sociopolitical environment. Another conceptual model of self-transcendence from nursing is based on a literature review and uses the process of concept analysis (McCarthy et al., 2018). The antecedents and attributes of self-transcendence are organized into five logically related domains: creativity, relationships, introspection, contemplation, and spirituality (Figure 2).

### 2.2. Phenomenological

There is a large body of theoretical literature and empirical research on self-transcendence. However, some of the conceptual models of self-transcendence mentioned above have had their internal validity questioned (MacDonald and Holland, 2002), suggesting that in when reporting self-transcendence an individual might not be able to separate core constructs; and the construct of self-transcendence itself needs further analysis (Akyalcin et al., 2008). There is less research on the process, outcomes, and nature of self-transcendence itself.

Garcia-Romeu et al. (2015) used a grounded theory approach to address shortcomings in the current understanding of self-transcendence in the experiential domain. In terms of context, self-transcendence likely occurs during stressful or challenging times in life, in adulthood, during religious/spiritual practices or social events like concerts/raves, and is catalyzed with psychoactive drugs, spiritual instruction, dance, and prayer. In terms of phenomenology, self-transcendence is described as very physical, whether that be warmth, connectedness, lightness, vibration or shaking, hyper-ventilating, nausea, and vomiting. Perceptually, there is an expansive change in self-boundaries, egolessness, and timelessness. Cognitively and affectively, participants experienced positive affective states (e.g., joy, love, compassion, forgiveness, wonder, and freedom), surrender, vulnerability, and openness. Many people revealed that words and the vehicle of the mind could not really explain their self-transcendent experience because the experience itself transcended such boundaries. In terms of aftermath, the short-term effects consisted of mainly decreased anxiety, increased energy, insight, socialability, and sustained positive affect. Long-term effects were related to enduring transformation and impact, i.e., worldview, self-concept, and value-orientation. The outcomes for aftermath were value re-orientation, increased concern for others, increased positive affect, and disidentification from old patterns of thinking or behavior. The authors recommended self-transcendence needs more research on the pathological manifestations and its potential role in promoting enhanced well-being.

Metzinger (2020) proposed non-dual awareness as a non-conceptual, minimal phenomenal experience. Non-dual awareness or consciousness-as-such is a highly related to self-transcendence, described by Josipovic (2019) as “an empty cognizance, aware and present, but without any thoughts, emotions or perceptions, without a sense of body, space, orientation, time or the usual sense of self” (p. 279). In other words, non-dual awareness is where the self and world are merged into a unified whole or the boundaries of the self are dissolved (Gyamtso, 2001). Non-dual awareness occurs during states of lucidity in sleep and absorption in meditation (Thompson, 2010).

Nour et al. (2016) conceptualize self-transcendence in the same way as Yaden et al. (2017a)—a disappearance of the sense of self. Nour et al. (2016) describe ego consciousness as having two main constructs. First, ego-dissolution as “the associated feeling of increased union with one's surroundings, known as dissolved ego-boundaries.” Second, ego-inflation as “the distinct and largely antithetical experience of unusually elevated self-assuredness and confidence.” Thus, self-transcendence would then be the presence of ego-dissolution and absence of ego-inflation.

### 2.3. Physiological

**Figure 3:**
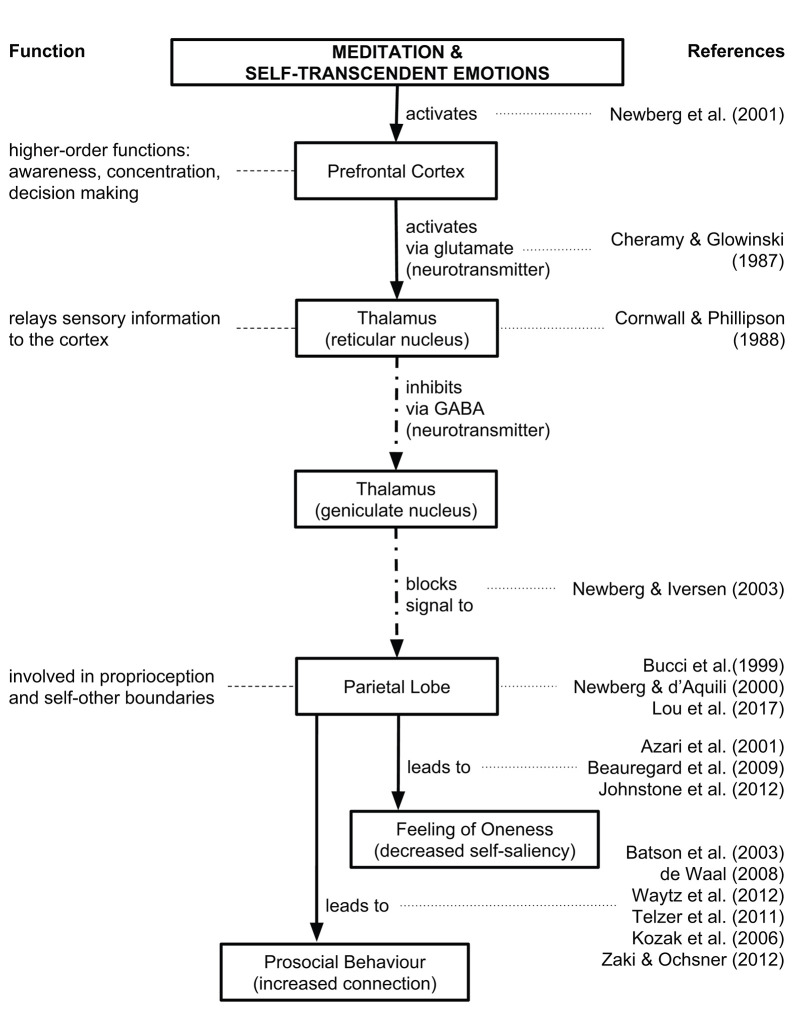
Neurological processes relating to self-transcendence. For a review, see (Newberg, 2014; Yaden et al., 2017a,b).

**Figure 4:**
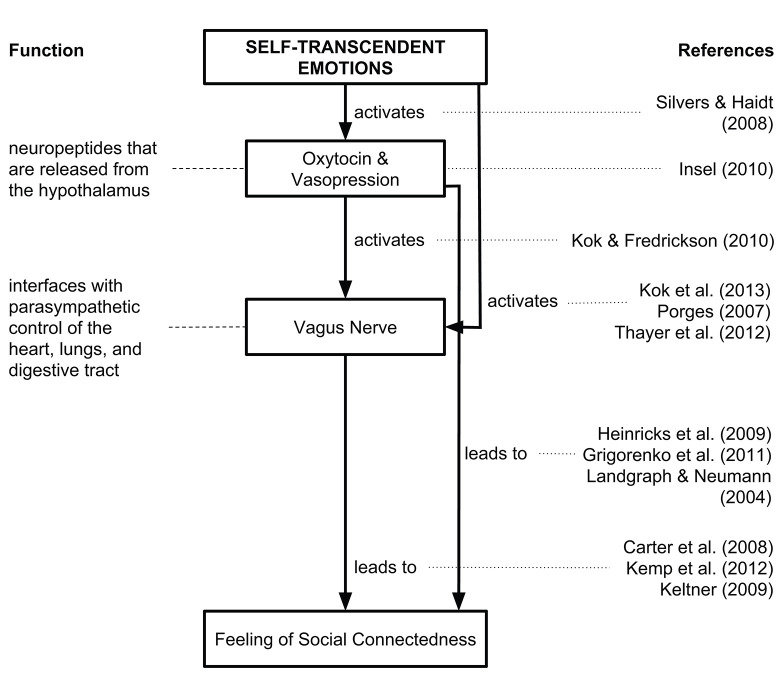
Biochemical processes related to self-transcendence. For a review, see (Yaden et al., 2017a).

Much of the neurophysiological research on self-transcendence relates to meditation and specifically mindfulness meditation, since, according to some, one component of mindfulness is self-transcendence (Vago and Silbersweig, 2012). There is also research on the neural correlates of experiences that are under the umbrella of self-transcendence such as awakening, enlightenment, and mystical experiences. We describe these studies as they relate to self-transcendence but there are always limits when the construct of self-transcendence highly varies across studies. For an overview of neurological and biochemical underpinnings of self-transcendence, see Figures 3, 4.

In studying *awakening experiences*, which de Castro (2017) describes as a transcendent experience or “an essential core experience of oneness,” he presents a model that postulates three processing layers: sensory, perceptual, and cognitive. An awakening experience occurs when these three layers are removed. Testing this model is challenging since scientific methods are difficult to apply to phenomena that cannot be reliably and repeatedly produced. Nevertheless, de Castro (2017) sought the specific neurophysiological underpinnings of self-transcendence by comparing his model to existing research. First is the default mode network (DMN), which consists of the medial temporal lobe (declarative or long-term memory), medial prefrontal cortex (analyzing and thinking about attributes of other individuals), posterior cingulate cortex (autobiographical memory), and the ventral precuneus (hub of DMN) as well as parts of the parietal cortex (proprioception) (Smallwood et al., 2012). DMN activity is reduced during contemplative practice, such as meditation (Hasenkamp and Barsalou, 2012; Berkovich-Ohana et al., 2013b; Garrison et al., 2014), as well as in one study showing a video of awe (van Elk and Rotteveel, 2019). This suggests that contemplative practice reduces activity in neural structures involved in cognitive processing. This restraint on the cognitive system in turn amplifies the perceptual and sensory systems. In fact, those brain areas related to perceptual and sensory systems were larger in meditators and cognitive brain areas were smaller (Fox et al., 2014). Specifically, Fox et al. (2014) found these meditation brain areas increased in size: rostrolateral prefrontal cortex (metaawareness and introspection), sensory and insular cortices (body awareness), and anterior, mid-cingulate, and orbitofrontal cortex (emotion regulation). Moreover, these DMN areas were reduced in size: posterior cingulate cortex (self-related thinking), angular gyrus (transfers visual information to Wernicke's area), precuneous (self-awareness), and temporoparietal junction (self-other distinctions). Second, lesion studies have also looked at how self-transcendence is affected pre- and post-surgery and found that damage to the posterior parietal lobe, which is related to body sense and bodily interrelationships, increased self-transcendence (Urgesi et al., 2010; Johnstone et al., 2012). Third, transcranial magnetic stimulation (TMS) of inferior parietal lobe, related to body perception and separation of the environment, increases religiousness and spirituality (Crescentini et al., 2016). Other methods for eliciting self-transcendence are direct electric stimulation of brain regions (Blanke et al., 2002, 2004) or transcutaneous vagus nerve stimulation (Finisguerra et al., 2019). Finally, psilocybin has been found to produce reliable effects indistinguishable from awakening experiences; shows decreased DMN and feelings of oneness. Some researchers have looked at studying individuals who can have transcendent experiences regularly, but this method has its flaws (see Davis and Vago, 2013). Another approach is to develop adequate model test conditions, i.e., better experimental procedures for evoking awakening experiences, such that we can better define the model.

Davis and Vago (2013) outline whether we can determine the specific neural correlates for *enlightenment*, a term often equated with awakening in Buddhist traditions. The short answer: no. They argue measuring enlightenment is not possible because it is too vaguely defined as a construct. There is too much disagreement, even within Buddhist traditions, on what states and traits define enlightenment over mere concentration. Even if they are agreed upon, people may not agree on whether that state was achieved or not. Even Buddhist teachers do not accept self-reports of meditation experience at face value, but rather assess practice history, the manner and emotional state the report is given, and retrospective observations of behavior. Despite these concerns and challenges, Davis and Vago (2013) offer a potential solution by integrating evidence from neuroimaging with evidence of behavioral transformations specified in particular traditional descriptions of meditation practice. To this end, neuroimaging techniques have shown potential neural correlates of enlightenment. Cessation, referring to cessation of all inward phenomena in meditation, is linked to increased activity in the frontal polar cortex or Brodmann area-10 (higher cognitive functioning) (Koechlin et al., 1999; Ramnani and Owen, 2004). However, there are limitations with fMRI because it relies on generalized linear modeling, which carries some assumptions that a low-resolution fMRI signal, state bleed-over, and subtle states of enlightenment might violate. EEG found gamma band power over the lateral frontal and parietal sites correlated with self-reported clarity in meditation practitioners, suggesting a particular mechanism for increased phenomenal intensity (Lutz et al., 2004).

In terms of the Self-Awareness, -Regulation, -Transcendence (S-ART) model, where “T” represents the self-transcendent aspect of mindfulness, there are brain regions for self-specifying (i.e., experiential enactive self and experiential phenomenological self) and self-related processes: the dorsal attentional system and hippocampal-cortical memory system (Vago and Silbersweig, 2012), both components of the DMN. The S-ART framework suggests mindfulness critically involves working memory, efficiency of memory encoding, retrieval, and extinction processes, all aspects of hippocampal and parahippocampal activity, which serve higher order cognitive functions. This relates back to de Castro (2017)'s model of awakening experience, suggesting a common mechanism behind these types of experiences.

One review on the psychological and neurobiological mechanisms that may mediate the effects of self-transcendence comes from Yaden et al. (2017a), who conceptualize self-transcendence as having two major components: loss of self and increased connectedness. In terms of the loss of self, they found superior and inferior parietal functioning is associated with representations of the body's state (i.e., self and other representations) (Newberg et al., 2001), and is decreased when people report having a mystical experience (Azari et al., 2001; Beauregard and Paquette, 2006; Johnstone et al., 2012). Lesions in parietal regions lead to disassociation with the self and out-of-body experiences (Urgesi et al., 2010). Thus, a self/other overlap may also relate to the pro-social qualities of self-transcendence. In terms of the increased connectedness, neurological correlates might be oxytocin and arginine vasopressin, which are associated with social connection (Landgraf and Neumann, 2004; Heinrichs et al., 2009; Grigorenko, 2011). However, the research on these neuropeptides is based on animal models, so the validity of these claims for humans is limited. Researchers also found the vagus nerve is activated during self-transcendent positive emotions such as awe, compassion, gratitude, and love (Keltner, 2009; Kok and Fredrickson, 2010; Thayer et al., 2012; Kok et al., 2013). The vagus nerve is a cranial nerve that interfaces with parasympathetic control of the heart, lungs, and bladder. Since a parasympathetic response is associated with a sedative state, the vagus nerve activation would decrease under “stress” and increase when the body is at rest or in a peaceful state, which could be associated with positive emotions such as self-transcendence.

While we have described a simplified model of the neurological processes related to self-transcendence in Figures 3, 4, a more comprehensive model is suggested by Newberg and Yaden (2018). These authors have put forth a recent review on the brain processes involved with altered-states of consciousness, which they define as a type of self-transcendent experience “associated with intense experiential components and are frequently interpreted in reference to religious and/or spiritual concepts.” Their model shows these major brain areas likely involved in altered-states of consciousness including the prefrontal and anterior cingulate cortex, the thalamus, the parietal lobe, the hippocampus and amygdala, as well as the hypothalamus and autonomic nervous system.

## 3. Literature Search Methods

We conducted a literature search using ACM, IEEE, and ProQuest databases to identify relevant research articles. We performed additional hand searches of references in the retrieved literature. Our search for studies tapping into the measurement of self-transcendence included articles and empirical work published until September 1, 2019. The search strategy considered only studies published in English. Primary search terms were “self-transcendence,” “mindfulness,” “flow,” “self-transcendent positive emotions,” “awe,” “peak experiences,” “mystical experiences,” “altered states of consciousness and transcendence” plus “measurement,” “assessment,” “questionnaire.” Secondary search terms included self-transcendence and the name of the scale spelled out or abbreviated. We excluded measures that did not fit the definition of self-transcendence as “a feeling of decreased self-saliency and increased connection.”

## 4. Results: Research/Evaluation Methods for Investigating Self-Transcendence

Methods of investigating self-transcendence can be divided into five major categories: questionnaires and surveys; diary and journal entry; interviews; neurological and physiological measures; and behavioral measures. Next, we will evaluate and discuss these methods with reference to the table in the Supplementary Material.

### 4.1. Questionnaires and Surveys

Many attempts have been made to capture the subjective nature of self-transcendence through self-report questionnaires. Defining self-transcendence is of utmost importance because, of course, we cannot compare results if researchers are using different definitions. On the other hand, self-transcendence can be looked at as having several facets; studying these different facets could be a way of looking at self-transcendence from multiple lenses, e.g., Yaden et al. (2017a). Next, we briefly describe questionnaires for each of these facets, and we further discuss the validity and reliability in section 5.2.

#### 4.1.1. Mindfulness Questionnaires

Mindfulness questionnaires can be divided into two different categories: state and trait. State mindfulness is a fluid or short-term mindset that is flexible in how you perceive the world; it is linked to a concurrent experience or transient mood. Trait mindfulness, on the other hand, is a more permanent facet of personality, most likely linked to genetics. A state, when repeatedly elicited, can often result in a trait change (Kiken et al., 2015). Most mindfulness questionnaires are aimed at assessing trait mindfulness: Mindful Attention Awareness Scale (MAAS) (Brown and Ryan, 2003), Freiburg Mindfulness Inventory (FMI) (Buchheld et al., 2001), Kentucky Inventory of Mindfulness Skills (KIMS) (Baer et al., 2004), Cognitive and Affective Mindfulness Scale (CAMS-R) (Feldman et al., 2006), Southampton Mindfulness Questionnaire (SMQ) (Chadwick et al., 2008), and Five-Facet Mindfulness Scale (FFMQ) (Baer et al., 2006). Capturing one's ability to get into a state is challenging, and there are different approaches. One approach is by using several facets to capture mindfulness. FFMQ and KIMS both have multiple facets to measure mindfulness, and report good internal consistencies, α = 0.89 and 0.76 − −0.91 respectfully. However, despite their perceived comprehensiveness, researchers have found the multiple sub-scales provide redundant information and the time to complete the surveys is long. MAAS, FMI, and CAMS-R all purposefully have one total score because the researchers believe mindfulness is comprised of many things that cannot be separated out into sub-scales. MAAS is the mostly widely used mindfulness scale, with an internal consistency of 0.82, test-retest reliability of 0.81, and adequate convergent and discriminant validity. FMI is the second most widely used mindfulness scale, but unlike MAAS it includes a focus on curiosity as a part of the definition of mindfulness; FMI has an internal consistency of 0.93. CAMS-R is the shortest holistic measure of mindfulness with only 12 items. Internal consistencies ranged from 0.74 − 0.80. Lastly, SMQ is different from all the other scales because it measures a mindful approach to distressing thoughts and images. SMQ has an internal consistency α = 0.89; significant correlation with MAAS *r* = 0.57.

There exist only two mindfulness questionnaires that aim to measure state mindfulness: State Mindfulness Scale (SMS) (Tanay and Bernstein, 2013) and Toronto Mindfulness Scale (TMS) (Lau et al., 2006). TMS contains two sub scales, curiosity and decentering, with internal consistencies of 0.93 and 0.91, respectively. SMS has also shown a high reliability of α = 0.95. While TMS seems to be more widely used, it is intended for meditators, so languages used may not be accessible to novices. SMS, on the other hand, was designed to be used with all levels of mindfulness meditation and does not use jargon terms.

Another questionnaire that aims to measure meditation regardless of practice type, i.e., mindfulness or otherwise, is the Meditation Depth Questionnaire (MEDEQ) (Piron, 2001). MEDEQ is widely used because of its meditation practice agnostic quality and has good convergent validity (0.64 − 0.93) as well as high internal consistency (α = 0.92).

#### 4.1.2. Flow Questionnaires

Flow seems to be assessed mostly in the context of either games or physical activity. While many designers and developers aspire to flow, few actually have a scientifically valid and reliable way to measure it. Csikszentmihalyi (1990) started to develop the Flow Scale using semi-structured interviews, but that scale did not seem to have high reliability estimates. Jackson and Marsh (1996) have developed two separate scales to measure flow, based off of Csikszentmihalyi's flow theory: Flow State Scale (FSS) (Jackson and Marsh, 1996), Flow State Scale 2 (FSS-2) and Dispositional Flow Scale 2 (DFS-2) (Jackson and Eklund, 2002; Hamari and Koivisto, 2014). These scales are very similar in the questions they ask and their theoretical underpinnings, with similarly strong content and construct validity and reliability estimates ranging from 0.81 to 0.90. The difference is in the application: FSS measures flow experienced within a particular event and DFS measures the frequency of flow experiences in a given event in general.

#### 4.1.3. Self-Transcendent Positive Emotions and Awe Questionnaires

Positive emotions might be assessed using popular emotion scales such as the Positive and Negative Affect Schedule (PANAS) (Watson et al., 1988), Self Assessment Manikin Scale (SAM scale) (Bradley and Lang, 1994), or International Affective Picture System (IAPS) (Lang et al., 1997). However, these do not focus on the specific positive emotions relating to self-transcendence, namely elevation, compassion, admiration, gratitude, love, and awe. There exist three scales that attempt to capture these emotions: Inclusion of Other in Self Scale (IOS) (Aron et al., 1992), Dispositional Positive Emotion Scales (DPES) (Shiota et al., 2006), and Modified Differential Emotions Scale (mDES) (Fredrickson et al., 2003). IOS uses a set of Venn Diagrams to assess self-other overlap with good test-retest reliability (*r* = 0.83 overall), and good convergent, discriminant, and predictive validity. DPES assesses seven different positive emotions that are closely related to self-transcendence, with good reliability estimate ranging from 0.75 to 0.92. However, DPES does not assess positive emotions directly, but more people's dispositions toward positive emotions. mDES measures 20 discrete emotions, including positive emotions amusement, awe, contentment, gratitude, hope, love, pride, sexual desire, joy, interest, surprise and eight negative emotions. mDES can be used as a reliable tool for the assessment of positive (α = 0.79) and negative (α = 0.69) emotions for a specific time frame, e.g., the past 24 h. Thus, mDES is a retrospective, rather than present acute state, method.

Awe is a specific self-transcendent positive emotion, and it is only relatively recently that researchers have attempted to create scales to capture awe. There are five awe questionnaires: The Nature of Awe Questionnaire (NAQ) (Shiota et al., 2007), Awe Experience Scale (AWE-S) (Yaden et al., 2018), Situational Awe Scale (SAS) (Krenzer, 2018), Awe and the Small-self (AS) (Piff et al., 2015), and Gratitude/Awe Scale (GrAw-7) (Büssing et al., 2018). Small-self is conceptualized by Piff et al. (2015) as “a relative diminishment of the individual self and its interests vis-a‘-vis something perceived to be more vast and powerful than oneself” (p. 2). NAQ and AS are the most widely used, mostly because AWE-S, SAS, and GrAw-7 have only been developed recently. NAQ is a reliable measure of the small-self (α = 0.82), but AS does not seem to have any studies mentioning reliability estimates. AS neglects the additional content suggested by treatments of awe conducted outside of psychology (e.g., admiration mixed with wonder and fear; sublime), whereas SAS reflects psychological, philosophical, and religious perspectives. Both SAS and AWE-S claim to be more robust measures of awe compared to NAQ and AS. AWE-S shows strong internal consistency for each of its six factors: altered time perception (α = 0.91), self-diminishment (α = 0.89), connectedness (α = 0.87), vastness (α = 0.85), physical sensations (α = 0.81), and need for accommodation (α = 0.80) (Yaden et al., 2018). AWE-S also demonstrated strong reliability (α = 0.93). A paper on validating the SAS showed good convergent validity compared to DPES-awe (Shiota et al., 2006) and AS (Piff et al., 2015); additionally there was adequate construct validity among the four factors: connection (α = 0.84), oppression (α = 0.83), chills (α = 0.81), and diminished self (α = 0.69) (Krenzer, 2018). The authors of SAS point out that while SAS and AWE-S share a similar factor structure, it differs in that AWE-S includes additional factors that did not emerge in SAS (i.e., perceived vastness and need for accommodation) and SAS includes a negatively valenced sub-scale of awe that may capture a broader range of contexts. GrAw-7 is an extended version of the 3-item Gratitude/Awe subscale of the Spiritual Practices (SpREUK-P) scale. It is intended to measure dispositional gratitude/awe as a trait, namely feelings of gratitude, reverence/awe, and experiencing the beauty in life. GrAw-7 is strongly correlated with the perception of the sacred in life (Daily Spiritual Experience Scale) in religious persons, but it can also be used in secular populations. One validation study showed it had good internal consistency (α = 0.82) (Büssing et al., 2018); however, no other study has confirmed this.

#### 4.1.4. Peak and Mystical Experience Questionnaires

Mystical experience, as conceptualized by Stace (1960) and summarized by Robertson (1962), is “the intuition of oneness with the ultimate spiritual reality” (p. 180). Some of the earliest research on self-transcendence aimed to objectively measure peak and mystical experiences in the context of peak performance, sport, religion and spirituality, hallucinogenic experiences. These scales are still relevant and used today. Two scales look at peak experiences: Peak Scale (PS) (Mathes, 1982) and Experience Questionnaire (EQ) (Privette and Bundrick, 1987). PS measures tendencies for peak experience, and not assessing peak experience themselves, whereas EQ explores the phenomenon of peak experience. Both PS (α = 0.94) and EQ (α = 0.70) have adequate reliability.

There are five scales that attempt to measure mystical experience: Mysticism Scale (MS) (Hood, 1975), Daily Spiritual Experience Scale (DSES) (Underwood and Teresi, 2002), Spiritual Transcendence Scale (Spirit-TS) (Piedmont, 1999), Mystical Experience Questionnaire (MEQ) (MacLean et al., 2012), and States of Consciousness Questionnaire (SOCQ) (Griffiths et al., 2006). Spirit-TS assesses mysticism as a personality trait with adequate reliability (α = 0.86). Spirit-TS generalizes to a wide range of faith traditions. MS and DSES both are used within the context of religious and spiritual experience, and report sufficient construct validity and internal consistency (α = 0.93). However, they may miss different noetic qualities of mystical experience because of their focus. SOCQ is similar to MS, expect more for hallucinogenic experiences. MEQ also assesses hallucinogenic effects but in laboratory settings. SOCQ contains 43 items from the MEQ, but adds distractor items to ensure whether a participant had a “complete” mystical experience or not. Both scales have shown good internal validity and reliability.

#### 4.1.5. Altered States of Consciousness and Transcendence Questionnaires

The questionnaires we have described so far have focused on specific constructs of self-transcendence. Here, we describe and discuss questionnaires that attempt to look at altered states of consciousness and transcendence generally. Three scales assess the occurrence of the phenomena on different consciousness dimensions: Phenomenology of Consciousness Inventory (PCI) (Pekala and Levine, 1982), Altered States of Consciousness Rating Scale (OAV) (Bodmer et al., 1994; Studerus et al., 2010), 5-Dimension Altered States of Consciousness (5D-ASC) (Dittrich, 1998; Dittrich et al., 2010), and 11-Dimension Altered States of Consciousness (11D-ASC) (Studerus et al., 2010). All of these scales are very lengthy (53 to 94 items) and have acceptable levels of reliability. They have been used mostly with understanding experiences from taking hallucinogens such as psilocybin. The consciousness dimensions themselves are different across these scales. 11D-ASC has better discriminant and convergent validity scores compared to 5D-ASC, but has lower reliability. The original OAV is shorter than the 5D-ASC with comparable reliability. The new OAV may provide a better fit, and lower order scales do have sufficient validity and reliability (42-items).

Two scales measure self-transcendence as non-dual awareness: Non-dual Awareness Dimensional Assessment-Trait (NADA-T) and Non-dual Awareness Dimensional Assessment-State (NADA-S) (Hanley et al., 2018). Both NADA-T and NADA-S are 13 items long with good reliability (*r* = 0.93). While, they have not been used widely in assessing self-transcendence since they are relatively new scales, they promise to capture aspects of self-transcendence that other measures do not. For example, TMS captures the decentering aspect of self-transcendence, while NADA measures the form and formless absorption (i.e., relational self-transcendence) and experiential emptiness of self (i.e., annihilational self-transcendence) (Hanley et al., 2018). Another consciousness related measure of self-transcendence is Ego-Dissolution Inventory (EDI)(Nour et al., 2016). EDI is 16 items that assess the associated feeling of increased union with one's surroundings through two constructs: ego-dissolution and ego-inflation. It has sufficient construct validity (ρ = 0.735), and is primarily used in measuring ego-dissolution in psychedelic experiences.

Other scales look at self-transcendence through different lenses: Self-transcendence Scale (STS) (Reed et al., 1989), Temperament and Character Inventory (TCI) (Cloninger et al., 1993b), Adult Self-Transcendence Inventory (ASTI) (Levenson et al., 2005), the Self Expansiveness Level Form (SELF) (Friedman, 1983), and the Portrait Values Questionnaire Revised-RR-Self-transcendence Subscale (PVQ-RR-ST) (Schwartz, 2012). STS, TCI, SELF, and PVQ-RR-ST measure self-transcendence as a trait, and not a state. STS is used widely in nursing studies and has been adapted for use with adolescent, adult, and older adult populations. Reliability ranges from 0.80 to 0.88, with test-retest reliability of 0.95. SELF is designed to assess self-expansiveness, which has been operationalized as three distinct levels based on a spatial-temporal cartography of self-concept: personal, middle, and transpersonal. A validation study found SELF to have good internal consistency (α = 0.66 − 0.81) and test-retest reliability (*r* = 0.8–0.83) (MacDonald et al., 1994). TCI has gone through several iterations. The original has 226 items with True/False answers and the revised TCI has 240 items using a 5-point Likert scale. Self-transcendence is a sub scale with 33 True/False items (TCI) or 26 5-point Likert scale items (TCI-R). SELF and STS are significantly shorter (15 items) but may not capture the subtle nuances of qualitative experience. TCI is comprehensive, but is not inclusive of religious and spiritual components of self-transcendence. ASTI measures transcendence as a developmental process that is more lifespan inclusive, and is used in studies assessing wisdom since self-transcendence is considered a high level of psychological development. Its reliability is satisfactory (α = 0.83), and moderate internal consistency of .66. PVQ-RR-ST is a sub-scale of the PVQ. PVQ is an alternative to the Schwartz Value Survey (SVS) that measures the ten basic values of persons not educated in Western schools that emphasize abstract, context-free thinking. It can be used with children as well as adult populations, and has good internal consistency (α = 0.76 − 0.85). Overall, the state scales seem to capture more the experience of self-transcendence, but have not been generalized across different experiences of self-transcendence. The trait and developmental scales of self-transcendence are good for assessing people's tendencies for self-transcendence, but may not be suitable for short-term measures.

### 4.2. Diary and Journal Entry

Diary studies, in general, are a research method for collecting information on participants' lives. Specifically, researchers are usually most interested in the behaviors, thoughts, and feelings of participants over time. Diary methods have been around for a long time, but modern diary methods are systematic and often highly structured. I will review three different diary methods: Narrative Recall, Diary Entries, and Experience Sampling.

Narrative Recall for studying emotion is when participants would recall and write about a personal experience involving the theoretically defined, prototypical elicitor of each emotion. Narrative Recall has been used in emotion research for decades as a well-validated measure (Shaver et al., 1987), however Griskevicius et al. (2010) further developed the method by recognizing that using the emotion word as a prompt was biasing participants and decreasing validity. With Griskevicius et al. (2010)'s method, participants are not asked to write about an “emotion word” so that the definition is not constrained by researchers; participants can give rich descriptions of positive emotions. Narrative recall has been used to capture both the state and trait experience of awe (Shiota et al., 2007; Piff et al., 2015; Yang et al., 2018; Zhao et al., 2018), and has been used in classifying emotions generally including self-transcendent emotions of love, compassion, amazement, wonderment, awe, and elation (Shaver et al., 1987). However, this method is very time consuming to both conduct and analyze. Diary Entries are long-term written accounts that are suitable for studying the long-term effect of an event, when the event cannot be studied in a laboratory, and when researchers want to know how participants reflect about an experience through time (Bolger et al., 2003). This method can give researchers new insight into a phenomenon and participants can give rich, thick descriptions. For example, researchers have used diary entries to study mindfulness, dreams, and mystical experiences (Hall and Van de Castle, 1966; Sokel, 1978; Cangas et al., 2008; Zhu et al., 2017). However, these accounts can be distorted, very selective and not representative of the whole experience (Janssens et al., 2018). For salient activities, they are less subject to retrospective bias than are interview data. Experience Sampling was one of the first psychology methods for studying emotions (Csikszentmihalyi et al., 1977) as a way to provide a valid instrument to describe variations in self-reports of mental processes. Participants report (e.g., write notes or type in an app) what they were experiencing just before the prompt of a pager or similar signaling device. The signals are typically given at random times to minimize instrumentation effects based on the expectation of a page at a particular time. The experience sampling method has been used in studying flow in different contexts: self-expansion in couples (Graham, 2008), experience at work (Fullagar and Kelloway, 2009), peak experience in the Grand Canyon (Panter, 2017), during sport (Jackson, 2000), and in virtual reality (Gaggioli, 2012). This method has also been used in studying different forms of focused attention or mindfulness (Easterlin and Cardea, 1998; Abuhamdeh and Csikszentmihalyi, 2012; Smallwood et al., 2012). Experience Sampling reports acceptable levels of internal consistency and test-retest reliability, but researchers recommend using other measures to corroborate the results and ensure validity (Csikszentmihalyi and Larson, 2014).

### 4.3. Interviews

Contemporary research on consciousness started with functionalist psychologists, phenomenology in philosophy, and the Gestalt approach. However, the popularity of behaviorism eclipsed this research until the 1960s with increased interest of altered states of consciousness (Cardeña and Pekala, 2014). Many scientists dismissed introspective methods claiming they were unreliable, biased, and un-objective. Others conceded that no science is observer-free because we always view science from a certain sociocultural and psychological perspective. Different interviewing methods of assessing introspective experience, such as self-transcendence, can be phenomenological, concurrent, or retrospective.

#### 4.3.1. Phenomenological Interviews

The interpretive framework in phenomenology is essentially postmodern: human experience is complex, experienced subjectively, and has meaning. Many articles on phenomenology research are focused on how the data are analyzed rather than how its obtained (Moustakas, 1994). Giorgi (1985), a phenomenology researcher, stated that questions should be broad and open ended so the participant can express their view point extensively and limit interviewer bias. However, he failed to describe how to proceed in the interview after stating those initial generalist questions. Bevan (2014) describes a three-part guide to phenomenological interviewing to help better consistency and address criticism of approach being too open ended and biased: contextualization, apprehending the phenomenon, and clarification. Phenomenological interviewing can provide thick, rich descriptions where the essence of experience or phenomenon emerges from participants rather than existing theory or research. Moreover, it does not impose existing theoretical models and is very comprehensive. However, it may be too structured for some researchers, an understanding of assumptions is required, philosophical ideas are abstract, participants need to be carefully chosen, finding participants may be difficult if the phenomena is very specific and rare, bracketing personal experiences is needed, and it is very time consuming. Phenomenological methods have been used to measure constructs related to self-transcendence such as lucid dreaming (Kitson et al., 2018), peak experiences (Panzarella, 1980), wonder (Gallagher et al., 2015), altered states of consciousness in float tanks (Kjellgren et al., 2004), meditation (Downey and Cohen, 2018); but also has been used to measure self-transcendence itself in women with breast cancer (Coward, 1990, 1991), gay men and women with AIDS (Coward and Lewis, 1993; Coward, 1995), and in healthy populations (Coward, 1996). Microphenomenology is a method for exploring lived experience very finely or for a singular event (Petitmengin, 2006), with a much more narrow view than phenomenology. This method is also comprehensive, with a rigorous technique, high level of reliability, and fine degree of granularity. However, the interviewer needs to be highly trained in the interviewing technique to ensure reliability. Microphenomenology has been used to study awe (Quesnel et al., 2018; Stepanova et al., 2019), meditative experiences (Petitmengin et al., 2017; Prpa et al., 2018; Przyrembel and Singer, 2018), and altered states of consciousness caused by DMT—a fast-acting tryptamine psychedelic (Timmermann et al., 2019). Both phenomenology and microphenomenology can be useful in describing self-transcendence, but which one you use depends on if you want to study the experience generally and across life times (phenomenology), or if you want to look at one specific experience in great detail (microphenomenology).

#### 4.3.2. Concurrent Interviews

Concurrent methods have the advantage of gathering rich data while the experience is happening, which significantly reduces memory bias that comes from retrospective reports. The drawbacks include being not easily quantifiable, it may not be a comprehensive account, there is a chance of reactivity during the process, and some experiences may not translate well because of temporal and representational limitations of language and translation between thought and experience. However, these methods seem to have good concurrent validity. Thinking Out Loud (Ericsson and Simon, 1980; Watson, 2009) is one method where participants verbally report what they are experiencing while they are experiencing it. This method can be helpful for real-time experiential reports, but can be intrusive or disruptive of the experience while it happens. Researchers have used the Thinking Out Loud method to understand experiences of mindfulness (Aslan et al., 2016), transformative experience in the context of diabetes (Paterson et al., 1999), and altered states of consciousness (Marcusson-Clavertz and Cardeña, 2011). Cued-recall Debrief (Omodei and McLennan, 1994), in contrast, allows the participant to first have the experience while they are recorded from the firs- person perspective, and then are asked to verbally report their thoughts and feelings while watching the replay. The major advantage of this is not disrupting the experience itself; this is especially important for a transcendent experience where disrupting the participant to ask questions would most likely negatively impact the experience. These methods have been used in several Human-Computer Interaction (HCI) and Psychology studies of emotion (see Supplementary Material for details). For example, the cued-recall debrief method has been used to assess real-time emotions (Bruun and Ahm, 2015; Bruun et al., 2016), affective responses (Bentley et al., 2005), and decision making (Omodei and McLennan, 1994). While this method seems promising in assessing states of self-transcendence in real-time, only a few studies have actually managed to implement this measure for self-transcendence specifically (Quesnel et al., 2018; Stepanova et al., 2019).

#### 4.3.3. Retrospective Interviews

Retrospective interviews are used when interviewing the participant during the experience is not possible or impractical. Often this is done with self-transcendent experiences that have already happened to the participant. The most common type of retrospective interview for self-transcendence is the semi-structured interview (Kvale and Brinkmann, 2015). The goal is to capture rich, detailed nuanced answers that uncover subjective differences and specificities of the interviewee. Participants verbally respond to questions that were created beforehand. Normally all of the questions are asked, and similar wording is used from interviewee to interviewee. However, sometimes questions may not always follow the exact order. While this allows more freedom to explore self-transcendence, which may also provide new insight, this method is time consuming and the quality of data largely depends on the interviewer's skills. Rather than open interviewing, which has virtually no structure, semi-structured interviews are pertinent when the researcher is beginning the investigation with a fairly clear focus rather than a general focus, so that they can address more specific issues. Structured interviews might be too restrictive for exploring a phenomenon like self-transcendence. Another form of retrospective interviewing is Retrospective Reports; Maslow (1959) provided a guide to investigating peak experiences where participants are asked to describe the most wonderful or joyful experience of their lives, how they felt, and if and how it affected their lives through in-depth interviews or open-ended surveys (15–30 min). Similar to semi-structured interviews, there are usually only a few pre-determined questions. However, retrospective reports include a coding scheme and member checking in the process to improve reliability and validity. Both semi-structured interviews and retrospective reports can provide rich detail and a comprehensive view on self-transcendence. However, both suffer from similar drawbacks of memory bias.

### 4.4. Neurological and Physiological Measures

There are a variety of physiological measures that tap into self-transcendent experiences. Many studies involve meditation, which is related to self-transcendence, but not much research is on self-transcendence in particular. There is a challenge with physiological measures mapping to specific states and emotions because our bodies are complex and the same physiological response (e.g., increased heart rate) may serve multiple functions and correlate with different states depending on other factors. That said, we can still use physiological measures as yet another lens into understanding self-transcendence. We will discuss two types of measures: neurological and non-neurological (which we will simply call physiological).

#### 4.4.1. Neurological Measures

Neurological changes in the brain can be observed through several techniques, each with advantages and disadvantages. There are two types of neurological techniques: direct and indirect measures. Direct measures look at neural activity of the brain itself, while indirect measures look at other bodily changes that are related to direct brain activity. Direct measures include brain lesions, Electroencephalography (EEG), and Magnetoencephalograph (MEG). Indirect measures are Positron Emission Tomography (PET), Single Photon Emission Computed Tomography (SPECT), Magnetic Resonance Imaging (MRI), Functional Magnetic Imaging (fMRI), and Functional Near-Infrared Spectroscopy (fNIRS).

Brain lesions are one of the oldest neurological methods for investigating the link between brain function and behavior (Broca, 1861). Participants have portions of their brain removed and a comparison is made between performance before and after the lesion and consequent deficits are noted. One can also study participants whose brains have been damaged through natural means. Typically, this method is now only used when participants need surgery on that particular part of the brain, since it would be unethical to run a true experiment. While this method shows us insight into the causally necessary function of brain structures, it is extremely invasive, often not generalizable, and assumes that discrete anatomical modules deal with different cognitive functions. Studies have shown lesions to the parietal lobe increase the propensity for self-transcendence (Urgesi et al., 2010). Another direct measure of brain function is EEG, which directly measures brain electrical activity just under the scalp via electrodes placed on the skin (Berger, 1929). This method is widely used to study experiences such as meditation and hallucinogens because it is non-invasive, portable, relatively low-cost, and provides good temporal resolution. Different meditation types may correspond with different brain waves: focused attention (beta: 13–30 Hz; gamma: 30–50 Hz), open monitoring (theta: 4–8 Hz), and automatic self-transcending (alpha-1: 8–10 Hz) (Cahn et al., 2012); although neuroelectric correlates of meditation have not yet been firmly established and should be considered with caution (Fox and Cahn, 2018). This method is good for recording real-time measures of brain activity, and several consumer EEG products make it accessible to researchers. However, there are several drawbacks to EEG. First, electric conductivity may vary widely from person to person and also over time, due to the natural conductivities of other tissues such as brain matter, blood, and bones. Because of this, it is sometimes unclear exactly which region of the brain is emitting a signal. Also, it can be difficult to determine the source of the underlying signal since EEG only records signals directly below the scalp. It also requires a reference electrode, which may not always be reliable. EEG has been used to measure meditation-induced altered states of consciousness (Lehmann et al., 2001; Aftanas and Golocheikine, 2002), mystical experience (Tenke et al., 2017), and near death experience during meditation (Beauregard et al., 2009). EEG has also been used to measure related constructs of self-transcendence including brain states during transcendental meditation (Banquet, 1973; Yamamoto et al., 2006), yogic meditation and trance (Das and Gastaut, 1955), and mindfulness meditation (Travis and Arenander, 1994; Kerr et al., 2013). The popularization of mindfulness meditation in Western culture and the development of consumer EEGs has stemmed several meditation-related digital experiences and apps that claim to measure mindfulness (Hinterberger, 2011; Choo and May, 2014; Prpa et al., 2015; Amores et al., 2016; Gervais et al., 2016; Kosunen et al., 2016; Gu and Frasson, 2017; Antle et al., 2018). A more recent technique that is similar to EEG is MEG, which maps brain activity by recording magnetic fields produced by electrical currents occurring naturally in the brain, using very sensitive magnetometers (Cohen, 1968). Here, participants may be positioned on a movable examination table or seated in a comfortable chair within a room that shields out any electric and magnetic noise that could interfere with the exam. They will be positioned within the stationary helmet that contains the MEG detectors placed on the head. MEG has been used to measure brain activity during transcendental meditation (Yamamoto et al., 2006), mindfulness meditation (Kerr et al., 2013; Wong et al., 2015), and mindfulness-induced selflessness (Dor-Ziderman et al., 2013) and altered states of consciousness (Berkovich-Ohana et al., 2013a). Compared to EEG, MEG has improved spatial resolution because magnetic fields are less distorted by bone (skull). Moreover, activity is localized with more accuracy compared to EEG and is reference free. However, MEG is not as good as fMRI at localizing activity. Some other drawbacks are that MEG needs specialized shielding to eliminate the magnetic interference, it requires highly sensitive instrumentation, and it is not portable. Both MEG and EEG meet high standards of reliability and validity.

In terms of indirect measures of brain function, PET illustrates where neural firing is taking place by injecting a small amount of radioactive tracer and taking a picture of the cerebral blood flow (Phelps et al., 1975). This method is important for understanding the role of various neurotransmitters in cognition, such as glutamate and GABA (Newberg et al., 2001). PET's advantages include little artifacts in the scan, high spatial resolution, and quick scan times (30 s). The disadvantages are that we can only locate generalized areas of brain activity and not specific locations, it is expensive, invasive, and not suitable for children or vulnerable populations because of the radioactive elements. PET has been used to measure meditation-induced altered states of consciousness (Herzog et al., 1990; Lou et al., 1999; Kjaer et al., 2002), spiritual experiences (Borg et al., 2003), mindful awareness (Karlsson et al., 2011; Hakamata et al., 2013), and psilocybin-induced altered states of consciousness (Vollenweider et al., 1997; Gouzoulis-Mayfrank et al., 1999). Similar to PET is SPECT, which uses a radioactive compound and gamma camera to render a 3D map of a person's body (including brain) (Kuhl and Edwards, 1963). While SPECT is relatively inexpensive compared to PET, it does have longer imaging times and has a high rate of false positives. SPECT can be used to complement any gamma imaging study, where a true 3D representation can be helpful, e.g., tumor imaging, infection (leukocyte) imaging, thyroid imaging or bone scan. It can be used to provide information about localized function in internal organs, such as functional cardiac or brain imaging. SPECT has been used to show how meditation-induced peak experience activates the prefrontal cortex (Newberg et al., 2001). It has also been used to measure brain states during religious and mystical states (D'Aquili and Newberg, 1993), hallucinogenic experiences from mescaline (Hermle et al., 1998), and ayahuasca (Riba et al., 2006; Sanches et al., 2016). Another indirect measure of brain functioning is MRI, which measures the change in blood flow related to energy level in brain cells by typically using the blood-oxygen-level dependent (BOLD) contrast, (Lauterbur, 1973). Participants are subjected to a powerful magnet that aligns the hydrogen nuclei of water atoms inside of their brain and can determine changes in the amount of brain tissue (structural MRI) or in the amount of blood flow (functional MRI). Typically, the more active a particular brain structure, the more blood flow it receives. Functional MRI (fMRI) uses the same basic principles as structural MRI, except that the former measures metabolic activity around anatomical structures whereas the latter takes 3D images of the anatomical structures themselves. Like MRI, fMRI has been used to quantify the volume of particular brain structures. fMRI can be used to produce activation maps showing which parts of the brain are involved in a particular mental process, whereas MRI only shows the structures. This means that MRI can be used to measure changes in volume or make comparisons between different subjects, e.g., meditation (reviews: Gotink et al., 2016; Afonso et al., 2020). fMRI has many advantages: non-invasive; poses little health risk; usable for all ages, including in-utero; wide spread availability; relatively low cost per scan; good spatial resolution; and better temporal resolution than other indirect neuroimaging methods, although not as good as EEG. The disadvantages are that fMRI is expensive, not portable, very noisy, and cannot be used to evaluate neurotransmitter systems. The noise aspect is particularly troublesome for studying self-transcendence because it can be difficult to get into this state when a giant, loud machine is distracting the participant. A study with transcendental meditators showed that the noise from fMRI strongly influenced both subjective and neurophysiological responses during meditation practice, calling into question the fMRI results (Travis et al., 2020). There is no agreed-upon average value for fMRI reliability. There are so many factors spread out across so many levels of influence that it is almost impossible to summarize the reliability of fMRI with a single value. fMRI is one of the more widely used methods of brain imaging for self-transcendence and its constructs. Researchers have used fMRI to capture brain regions that are active during meditation (Lazar et al., 2000; Wang et al., 2011), spiritual experiences (Miller et al., 2019), near death experiences (Beauregard et al., 2009), admiration and compassion (Immordino-Yang et al., 2009), awe experiences (van Elk et al., 2019), and drug-induced altered states of consciousness with DMT (Daumann et al., 2010), psilocybin (Lebedev et al., 2015; Carhart-Harris et al., 2018), LSD (Carhart-Harris et al., 2016; Tagliazucchi et al., 2016), and ayahuasca (de Araujo et al., 2012; Palhano-Fontes et al., 2015). fNIRS is a non-invasive and safe optical technique that uses light emitting diodes or laser diodes to measure human cerebral cortex oxygenation changes in response to certain stimuli/tasks. Compared to fMRI, fNIRS is silent and tolerable to movement artifacts, it measures oxygenated hemoglobin (O_2Hb) as well as deoxygenated hemoglobin (HHb), it allows long-time continuous measurements as well as repeated measures in a short time span, and it has a higher temporal resolution. Some of fNIRS's limitations are that it has poor spatial resolution, little standardization, and can have unstable accuracy between sessions. Overall, fNIRS is good for when you want a small, wearable device that is movement robust, especially for neuromonitoring and neurorehabilitation. In the context of self-transcendence, fNIRS has been used to measure positive emotions such as awe, gratitude, and love (Hu et al., 2019), brain states during mindfulness meditation (Gundel et al., 2018), sustained attention meditation (Zheng et al., 2019), and drug-induced altered states of consciousness with psilocybin (Scholkmann et al., 2019).

#### 4.4.2. Physiological Measures

There are many techniques for measuring physiological processes related to self-transcendence. Psychophysiological measures use indices of bodily responses that reflect variation in psychological states (Potter and Bolls, 2012). Unlike direct measures of the brain, psychophysiological correlations are not causal, but are instruments in which to test theories. Psychophysiological techniques use specific tools for measuring the physiological response: GooseCam for philoerection (goosebumps) (Benedek et al., 2010); Pneumograph for respiration (Marey, 1878); Electrodemograph (EDG) for skin electrical activity (Vigouroux, 1879); Photoplethysmograph (PPG) for blood flow changes (Hertzman, 1937); Electrocardiograph (ECG/EKG) for heart rate and heart rate variability (HRV) (Einthoven, 1895); Electromyograph (EMG) for muscle electrical activity (Hardyck et al., 1966). Each of these psychophysiological instruments allows measuring specific characteristics of self-transcendence that would otherwise not be captured in self-reports (Bartholow and Bolls, 2013). For example, a study of awe showed a mixed valence by means of sEMG and a unique physiological pattern resembling a freezing or dissociation-like response (Chirico et al., 2017).

GooseCam investigates goosebumps as a response to strong emotional experiences by using a high-quality camera to record the surface of the skin. Goosebumps have been linked to constructs of self-transcendence such as peak experiences and awe (Grewe et al., 2009). Several research groups have used goosecam to measure instances of awe, e.g., (Benedek et al., 2010; Sumpf et al., 2015; Wassiliwizky et al., 2017; Quesnel and Riecke, 2018). This method is shown to be robust to variations in skin characteristics, but there is reduced internal validity because this method does not distinguish between the subjective sensation of chills and the objective phenomenon of visible piloerection. Pneumograph is one of the earliest physiological measures that records velocity and force of chest movements during respiration. Participants wear one or more straps around their chest and abdomen, which measures the expansion and contraction (i.e., volume) of their breathing. Respiration rate has been shown to correlate with level of mindfulness, i.e., slower breathing indicates higher degree of mindfulness (Ahani et al., 2014; Wielgosz et al., 2016), while showing mixed response to peak experiences and self-transcendent emotions, i.e., indicating both physiological arousal and physiological calming effects (Mori and Iwanaga, 2017; Clayton et al., 2019). While this is a relatively easy method to use, movement artifacts can influence the results. EDG measures skin electrical activity directly (skin conductance and skin potential) and indirectly (skin resistance) using electrodes placed over the digits or hand and wrist. As sweat increases in the ducts, the resistance at the skin goes down, resulting in higher levels of recorded skin conductance. Skin conductance is a reliable measure of emotional arousal that reflects the level of activation within the emotional and motivational systems. Skin conductance has been used as a measure of awe (Grewe et al., 2009; Chirico et al., 2017), peak experiences (Mori and Iwanaga, 2017), self-transcendent positive emotions (Shiota et al., 2011), and self-transcendence generally (Clayton et al., 2019). One thing to keep in mind when using EDG is that it only measures emotional arousal, not valence. PPG measures the relative blood flow through a digit sensor attached by a Velcro band to the fingers or to the temple to monitor the temporal artery. PPG signal is strong and robust, but a huge range in individual differences and indirect measures of self-transcendence makes reliability challenging. A more direct measure of heart activity is ECG/EKG, which measures the electrical activity of the heart by using electrodes placed on the skin. ECG is more accurate than PPG in measuring HRV, however wearing electrodes on the torso, wrists, or legs can feel more invasive, especially if the participant has lots of hair. Heart activity in general is innervated by both the central and peripheral nervous system, so it can be difficult to determine the nature of the feeling, i.e., whether it is positive or negative. Another drawback of using HRV is that one must account for baselines and individual differences. HR and HRV have been used as measure of self-transcendence (Clayton et al., 2019), awe (Grewe et al., 2009; Sumpf et al., 2015; Wassiliwizky et al., 2017), peak experiences (Mori and Iwanaga, 2017), mindfulness (May et al., 2016), and self-transcendent positive emotions (Shiota et al., 2011). Finally, EMG, i.e., surface EMG, measures the electrical signal associated with muscle activity. In terms of emotion research, this is typically done by placing electrodes on specific facial muscles involved in the valence of emotional processing: corrugator supercilii, orbicularis oculi, and zygomaticus major. Facial EMG is a reliable and valid method for measuring emotional valence (Potter and Bolls, 2012). fEMG has been used to measure self-transcendent emotions (Clayton et al., 2019), awe (Chirico et al., 2017), and flow (Cheron, 2016). EMG can be a useful diagnostic tool, the electrodes are easy and quick to apply with minimal discomfort. Yet, adipose tissue (fat) can affect EMG recordings, compliant skin is needed for accurate readings, and muscle cross talk can occur.

All of these physiological measures can provide an objective view of self-transcendence because it is hard to fake physiological data. That said, the Hawthorne effect can threaten internal validity, i.e., people behave differently when they know that they are being observed. There is also an external validity threat when using a convenience sample, which is not representative enough of the population, because there is a huge variation in individual differences and a large, representative sample is needed to account for them. Correct sensor placement is also critical because wrong placement will result in measuring the incorrect bodily response. Most transcendence related research that uses physiological measures is actually measuring arousal and valence generally, and not a discrete emotion. This is because our bodily reactions are complicated and intertwined. However, there are some researchers trying to measure discrete emotions, such as awe, gratitude, and love (Koelstra et al., 2012; Shiota et al., 2017).

### 4.5. Behavioral Measures

Behavioral measures are overt actions and reactions that are observed and recorded, exclusive of self-reported behavior. Behavioral measures do not suffer the same reliability and validity threats that introspective measures do. However, self-transcendence is generally considered a psychological phenomenon, so studying it from a behaviorist perspective is difficult. Proxy measures look at aspects thought to be related to self-transcendence, such as pro-sociality and empathy, e.g., (Van Lange et al., 1997; Saroglou et al., 2008; Rosenberg et al., 2013; Piff et al., 2015; Quesnel et al., 2018). Other proxy measures include body size estimation (van Elk et al., 2016) and time perception (Rudd et al., 2012), where a smaller size estimation and slower time perception were correlated to self-transcendence. Although, a recent paper found experimentally induced awe does not affect implicit and explicit time perception (van Elk and Rotteveel, 2019). The main method of studying self-transcendence behavior is observation, where the researcher watches (i.e., observes) behavior as it organically and spontaneously unfolds in a natural environment. No one really knows when the method of observation for scientific study dates back to. In modern times, it is used in grounded theory and ethnographic approaches (Creswell, 2012). Participants are either aware (overt) or not (covert) they are being studied. Observations can be in a controlled setting, in a natural environment, or the researcher can join in as part of the group. Observational research is used when other data collection procedures, such as surveys, questionnaires, etc. are not effective or adequate. They are also used when the goal is to evaluate an ongoing behavior process, event, or situation; or when there are physical outcomes that can be readily seen. For example, gestures, interactions, voice qualities, and facial features like eye gaze, pupil dilation, and blinking can indicate an affective state (Sharma and Gedeon, 2012). Typically, these behaviors are coded and analyzed across data sources. Observations can explain meaning and context, which is important for self-transcendence. However, they can be viewed as too subjective, the Hawthorne effect can affect validity, and results depend on the researcher's role. Overt observation may affect validity of findings, and covert may be unethical, have a high potential for role conflict, and may not tell the whole story. This method is also very time consuming and, in the case of self-transcendence, should probably be used in conjunction with other methods rather than relying on a single data source. Some researchers have used observation in studying awe (Quesnel et al., 2018), mindfulness (Gervais et al., 2016; van Rooij et al., 2016; Antle et al., 2018), self-transcendence in women with breast cancer (Coward, 1990), and ego dissolution after taking ayahuasca (Uthaug et al., 2018).

## 5. Discussion

### 5.1. Conceptual Challenges of Self-Transcendence Methods

#### 5.1.1. Different Underlying Theories for Different Measures

Generally, there are three views of self-transcendence: physiological (neurobiological mechanisms), phenomenological (experiential), and conceptual (traits, values). Each of these theories offer a different perspective in which to define self-transcendence, which in turn impacts how one measures self-transcendence.

Physiological measures of self-transcendence aim to objectively capture states of self-transcendence. For example, people report having a mystical experience when there is decreased activation in parietal functioning (Azari et al., 2001; Beauregard and Paquette, 2006; Johnstone et al., 2012). That said, even seemingly objective measures are not directly capturing self-transcendence as a whole but are instead measuring correlates or outcomes of self-transcendence, e.g., increased heart rate.

Phenomenological measures of self-transcendence aim to capture the lived experience of self-transcendence as a whole and highly individualized phenomenon, e.g., through ground theory interviews (Garcia-Romeu et al., 2015). Whereas physiological measures are reductionist, phenomenological measures of self-transcendence seek to understand the context in which self-transcendence occurs, the physical sensations, and the perceptual, cognitive and affective experiences.

On the other hand, conceptual measures of self-transcendence consider a wider range of social, developmental, and personality facets of self-transcendence that physiological and phenomenological measures do not capture. Still, conceptual measures should be used with caution since they are each developed from a specific theoretical lens. For example, self-transcendence as a personality trait in Nursing is derived from Neo-Piagetian theories. Moreover, when looking at the mindfulness facet of self-transcendence, these measures are derived from either Buddhist psychology (e.g., FMI) or with a pragmatic approach (e.g., KIMS) (Sauer et al., 2013). There are other conceptually based measures of self-transcendence that view it as a state, not a personality trait, e.g., TCI (Cloninger et al., 1993b).

#### 5.1.2. Unclear Constructs and Facets of Self-Transcendence

Koenig (2008) postulates that the term “spirituality” is often conflated with general good mental well-being or health. This is problematic both because the construct should be distinct and because this definition deviates from the original meaning of the word, i.e., religious or secular person living by a set of ethical and moral values and meanings. Self-transcendence can also suffer from this same issue. For example, several measures only consider the positive aspects of self-transcendence and exclude the “dark side,” including absorption, suggestibility, and dissociation, which can have psychotic outcomes (MacDonald and Holland, 2002).

The term “self-transcendence” is so broadly defined in the literature that it can encompass a wide range of constructs that each contain multiple facets. For example, mindfulness is considered a construct of self-transcendence (Yaden et al., 2017a), and mindfulness itself has several facets, e.g., FFMQ (Baer et al., 2006). With this broad and widely encompassing definition, it is difficult to derive a measure that is specific to self-transcendence and at the same time encompasses all its aspects.

#### 5.1.3. Direct Measures vs. Outcomes and Correlates of Self-Transcendence

Self-transcendence in itself can be difficult to capture and measure, especially because re-creating the right conditions for a self-transcendence to occur is challenging. Some methods ask the participant to recall a time when they had a self-transcendent experience, e.g., AWE-S (Yaden et al., 2018) or EQ (Privette and Bundrick, 1987), while others try to evoke the experience, e.g., micro-phenomenology (Petitmengin, 2006). In order to avoid having to reproduce self-transcendence or better tap into a previous experience, researchers have looked at proxy measures. For example, self-transcendence is reported to be correlated with self-diminishment (van Elk and Rotteveel, 2019) and pro-social tendencies (Carlo and Randall, 2002). These proxy measures can be powerful tools when direct measures cannot be used or are nor feasible. However, researchers need to be clear on what exactly they measure—self-transcendence or its outcome.

### 5.2. Empirical Support of Self-Transcendence Methods

#### 5.2.1. Internal Consistency Reliability

One measure of internal consistency (reliability) is Cronbach's alpha. α ≥ 0.70 is considered acceptable in most social science research situations. We found that the questionnaires all had sufficient alpha. However, the proxy measures were still lacking measures of internal consistency, with the exception of the Prosocial Tendencies Measure (PTM) (Carlo and Randall, 2002) that showed high internal consistency of α = 0.92. The differences in high and low alpha scores could be the difference in narrow and broad scope. Questionnaires measuring specific constructs of self-transcendence will have higher alpha coefficients than those measuring self-transcendence more broadly. Additionally, those questionnaires with a higher number of items will have higher alphas. As we can see in Table 1, the more broad measures of altered-states of consciousness and self-transcendence, positive emotions, as well as proxy measures have lower alphas than the specific measures of awe, flow, mystical or peak experiences, and mindfulness.

**Table 1:** Reliability coefficients of internal consistency measured with Cronbach's alpha and test-retest measured with Pearson's r, and number of items for questionnaires that measure general self-transcendence (left) and specific constructs of self-transcendence (right).

**Questionnaire**	**Item #**	**α**	**r**
**General measures**			
ASC/ST			
PCI	53	0.75–0.82	0.34–0.56
OVA	66	0.73–0.91	0.77–0.83
5D-ASC	72	0.93	0.56–0.71
11D-ASC	94	0.7–0.8	–
STS	15	0.8–0.88	0.55–0.78
SELF	18	0.66–0.81	0.8–0.83
TCI	240 (33)	0.9	0.52–0.82
ASTI	18	0.66–0.83	–
PVQ-RR-ST	15	0.76–0.85	–
EDI	16	0.80	0.74
NADA-T	13	–	0.93
NADA-S	13	–	0.94
Positive emotions			
IOS	7	–	0.83
DPES	39	0.75–0.92	–
mDES	20	0.75	–
Proxy Measures			
ZTPI-F	13	0.78	0.63-.84
Small Self	–	0.51	–
PTM	26	0.92	0.56-.82
Meditation Depth			
MEDEQ	30	0.92	0.64-.93
**Specific Measures**			
**Questionnaire**	**Item #**	α	*r*
			
Awe			
NAQ	16	0.82	–
AWE-S	30	0.92	–
SAS	18	0.84	–
AS	13	0.92	–
GrAw-7	7	0.82	–
Flow			
FSS	36	0.72–0.91	0.748-.978
DFS-2	36	0.81–0.9	–
Mystical/Peak Experiences			
PS	70	0.92–0.94	–
EQ	47	–	0.7
MS	32	0.93	–
DSES	16	>0.9	0.85
Spirit-TS	24	0.86	–
MEQ	30	0.93	0.81
SOCQ	100	0.71–0.95	–
Mindfulness			
MAAS	15	0.82	0.81
FMI	30	0.93–0.94	0.895
KIMS	39	0.76–0.91	0.65-.86
CAMS-R	12	0.74–0.8	–
SMQ	16	0.89	–
FFMQ	39	0.72–0.92	0.66–0.86
SMS	23	0.95	0.55–0.65
TMS	13	0.91–0.93	0.66–0.73

#### 5.2.2. Test-Retest Reliability

Test-retest reliability determines the consistency of a test taken multiple times, often denoted as *r*. Often, *r* ≥ 0.7 is the accepted value, but this will depend on the time between test and retest, the length of the test, what is being measured, and the characteristics of the sample. We found that test-retest reliability has been infrequently evaluated in measures of self-transcendence. Approximately half of all the questionnaires tested for test-retest reliability (see Table 1). Of those, most questionnaires reported adequate test-retest reliability. Those with low test-retest reliability may be due to the nature of the test; trait measures of self-transcendence will be more stable over time compared to state measures. Test-retest reliability is an important psychometric property for establishing stability of these measures over time. We suggest more validation studies are needed to establish the stability of the construct.

#### 5.2.3. Validity

We considered four types of validity: construct, content, face, and criterion.

The questionnaires of self-transcendence that report adequate validity as the following: PCI, STS, SELF, TCI, ASTI, IOS, PTM, MEDEQ, AWE-S, SAS, DFS-2, EQ, MS, MEQ, SOCQ, MAAS, and TMS. Those that specifically mention construct validity, which can be a measure of overall validity, are PCI, STS, AWE-S, DFS-2, EQ, MS, MEQ, and MAAS.

Diary and journal entry methods overall have good construct validity and they have been used in emotion research for decades as a well-validated measure. Interview measures have good validity when rigorous technique is applied and they can also bring richness to the data that would not otherwise have been captured in a questionnaire or physiological measure. Observations too can have strong validity and bring an in-depth understanding when they are natural.

Neurological measures of self-transcendence vary in accuracy, and not many have been specifically used to measure self-transcendence, but have been used to measure mindfulness meditation and self-transcendent emotions, including decreased self-saliency and increased connection (see Figure 3). If considering self-transcendence as an emotional state, then neuroimaging can be limited since there is little evidence that emotions are correlated directly to one specific brain region (Lindquist et al., 2012). As we can see in Figure 3, self-transcendence spans multiple brain regions and networks, making it difficult for some neuroimaging techniques with low spatial resolution to accurately measure self-transcendence. For example, EEG and MEG have poor spatial resolution while having a relatively high temporal resolution compared to fMRI. Thus, capturing the specific brain areas of self-transcendence would be more accurate with fMRI. However, capturing self-transcendence in a large noisy machine where the participant is supine also comes with its own challenges.

Physiological measures of self-transcendence are also not straightforward because their indices are often simply emotional arousal and valence; it is difficult to correlate a single physiological response with a complex emotion such as self-transcendence. That said, when combined with other measures that provide ground truth, such as interviews, they can be good indicators that a change has occurred from a normal state of consciousness to an altered one. One research group showed that one can represent subjective experiences of emotion as categorical somatotopic maps (Nummenmaa et al., 2014). While physiological measures can accurately measure arousal, those seeking to find a measure of self-transcendence should use these physiological measures with caution. It is the opinion of some researchers that there is no physiological “finger print” of any emotion and that our efforts to measure emotions should be directed “to observe, map, and better understand the breadth, nature, and function of this variation in emotion categories” (Siegel et al., 2018, p. 36).

### 5.3. Recommendations

Having reviewed the literature on measures of self-transcendence, we have some recommendations on which measure to use to asses self-transcendence. That said, the answer depends on the research context and purpose. Since self-transcendence is broadly defined and contains many constructs, we suggest taking a multi-method approach and triangulating the results for increased validity. One existing methodology that seemed most aligned with capturing the nature of self-transcendence is neurophenomenology—the synthesis of neuroimaging to provide rich empirical data (e.g., EEG, MRI, PET) and valid descriptions of first-person subjective experience (e.g., phenomenology). Neurophenomenology was first introduced by Laughlin et al. (1992), and Varela (1996), and developed by Maturana and Varela (1991), Varela (1996), Thompson (2010). For the phenomenological component, Husserl (1983) developed a method of bracketing knowledge in order to focus on the experience itself and provide an unbiased account. Varela used this interview method to obtain reliable descriptions of first-person experience. Another interpretation of neurophenomenology is the use of micro-phenomenology—a qualitative, second-person interview for the research of first-person accounts of lived experiences. Micro-phenomenology was first introduced by Pierre Vermersch as “entretien d'explicitation” (explication interview) (Vermersch, 1994) and further developed by Petitmengin (2006); it is now becoming a central element of Varela's neurophenomenology. Taking from neurophenomenology, we suggest two broad approaches of assessing and investigating self-transcendence: (1) in-the-moment and (2) as a value, developmental process, or personality trait.

#### 5.3.1. Assessing and Investigating Self-Transcendence in the Moment

We propose combining some of the following methods for investigating self-transcendence in-the-moment, which we will elaborate on subsequently:

**Questionnaires and Surveys**

- Awe Experience Scale (AWE-S);
- Toronto Mindfulness Scale (TMS);
- Non-dual Awareness Dimensional Assessment Scale-State (NADA-S);
- Inclusion of Other in Self Scale (IOS);
- Dispositional Positive Emotion Scales (DPES);
- Mysticism Scale (MS)

**Observation**

- Observation

**Interviews**

- Micro-phenomenology

**Neurological and Physiological Measures**

- Electroencephalography (EEG);
- Function Near-Infrared Spectroscopy (fNIRS);
- Electrodemograph (EDG);
- Facial Electromyograph (EMG)

**Diary and Journal Entry**

- Diary Entry

**Questionnaires and Surveys**

From the list of questionnaires previously described, we suggest the Awe Experience Scale (AWE-S), Toronto Mindfulness Scale (TMS), Non-dual Awareness Dimensional Assessment Scale-State (NADA-S), Inclusion of Other in Self Scale (IOS), Dispositional Positive Emotion Scales (DPES), and Mysticism Scale (MS). To measure state self-transcendence, specifically the decreased self-salience component, we propose using the AWE-S, TMS, and NADA-S. There are very few questionnaires that measure state qualities to begin with, and what exist currently are often not well-validated. AWE-S, although new, already has some validation studies to support it and likewise for TMS and NADA-S. All of these scales measure a specific aspect related to self-transcendence and may not be all encompassing of the experience, but perhaps with both they will fill in the gaps until we have a better state measure for self-transcendence. Lastly, IOS might be useful in measuring the connectedness aspect of self-transcendence. This measure is unique in that it uses pictures instead of language, which might be useful for capturing the ineffable aspects of self-transcendence. IOS, TMS, and AWE-S are good for assessing “at the present moment,” so it might be useful to do pre- and post-measures of these three scales to see if there is a change. Although DPES and MS are not explicitly measuring self-transcendence in the moment, past life experience can play a huge role in both if self-transcendence occurs and how it is experienced. DPES can provide an assessment of participants' pre-disposition to self-transcendence and MS can provide a brief history of past self-transcendent experiences. With these two measures, one might better control for individual differences. Both of these questionnaires have good reliability and validity, and are widely used. Other questionnaires that assess pre-disposition or traits are not as inclusive for all types of self-transcendence, are not well-validated, or are very long and redundant.

**Observation**

Although psychometric tests can be helpful in understanding complex inner states such as self-transcendence, they often fail to capture the rich, qualitative experiential aspects. Observation methods are useful in filling this gap to see what people experienced both leading up to and during a self-transcendent experience. We suggest a setting where the participant has consented to be recorded but the researcher is not present so the participant can experience self-transcendence without the Hawthorne effect and at the same time researchers can more ethically collect their data. An example might be in a natural environment such as a meditation retreat. A lab could suffice and bring more control to the study, but we recommend creating a welcome and warm space rather than the usual cold and sterile conditions of a lab since mindset and setting are important.

**Interviews**

Since there are so many facets to explore with self-transcendence, a rich qualitative description would nicely complement the quantitative questionnaires and provide detail and context to the observations. We suggest using the micro-phenomenology method in conjunction with neurological and physiological measures we will describe in the next section. Micro-phenomenology seems to be a good fit for studying self-transcendence in this case because it allows the participant to fully experience self-transcendence without disturbing them with think-aloud methods. It also attempts to avoid memory bias by situating the participant back in the experience they just had through evocation. In contrast, a phenomenological interview seems more fitting to describe multiple experiences over time, whereas micro-phenomenology is about one specific event. Researchers still need to be cautious when using this method since the definition of self-transcendence is still quite broad. That is, there is a risk of missing a critical component while looking too deeply at one component of self-transcendence. Other retrospective interviewing methods seem more flawed and prone to memory bias compared to micro-phenomenology. Cued-recall debrief tries to address the issue of memory bias by showing a first-person recording of the experience during the interview, yet does not have the evocation element that makes micro-phenomenology potentially more valid.

**Neurological and Physiological Measures**

We would recommend to select some, or if feasible all, of the following neurological and physiological measures in conjunction with micro-phenomenology: EEG, fNIRS, EDG, and facial EMG. Most neurological measures are indirectly measuring something or are very expensive and not accessible, e.g., fMRI and MEG. Of all the neurological measures, EEG and fNIRS seem to be the best methods in this case because they are non-invasive, relatively inexpensive, portable, and are complementary measures of brain activity. EEG is already extensively used in studying related experiences such as meditation, so it seems like a natural extension to study self-transcendence. However, because of EEG's limited spatial resolution, it would be better to use fNIRS, which has a higher spatial resolution, in conjunction with EEG to achieve a more accurate measure of brain activity. In order to corroborate these measure of brain activity, we suggest using both EDG and facial EMG to measure emotional arousal and valence, respectfully. Both are required to get an accurate representation of the participant's affective state. Other physiological measures could be used to further corroborative the results. Still, it is preferable to not have to subject participants to too many electrodes as this can be uncomfortable, unnecessary, and maybe not all that more helpful.

**Diary and Journal Entry**

The last measure we would consider for investigating self-transcendence is diary entry. Self-transcendence can be a powerful and profound experience, but the real benefits continue well after the initial experience. With self-transcendence, one hopes to have a general sense of increased well-being, pro-sociality, and positive social values. Therefore, having participants complete diary entries would show how they reflect about the experience through time and show if and how a self-transcendent experience made a lasting and significant impact on their lives.

#### 5.3.2. Assessing and Investigating Self-Transcendence as a Value, Developmental Process, or Personality Trait

In this section, we suggest methods that are appropriate for measuring self-transcendence as a value, developmental process, or personality trait. We propose the following methods for investigating self-transcendence in their respective contexts based the their validity, reliability, suitability, and weighing the advantages and disadvantages:

**Value**

- Portrait Values Questionnaire Revised-RR-Self-transcendence Subscale (PVQ-RR-ST)
- Semi-structured interviews

**Developmental Process**

- Self-transcendence Scale (STS)
- Adult Self-Transcendence Inventory (ASTI)
- Diary Entries
- Retrospective Interviews

**Personality Trait**

- Dispositional Positive Emotion Scales (DPES)
- Temperament and Character Inventory (TCI)
- Mindful Attention Awareness Scale (MAAS)
- Dispositional Flow Scale 2 (DFS-2)
- Gratitude/Awe Scale (GrAw-7)
- Spiritual Transcendence Scale (Spirit-TS)
- Magnetic Resonance Imaging (MRI)
- Computed Tomography (CT)
- Experience Sampling
- Semi-structured Interviews

**Value**

Values are generally described as stable, broad goals that are important to us in life (Schwartz, 2012). Values express a person's motivations which may or may not be reflected in behavior. Thus, behavioral, observational, or physiological measures would not be suitable for measuring self-transcendence as a value. PVQ-RR-ST appears to be the only questionnaire measuring self-transcendence as a value. Here, values relate to universalism and benevolence, specifically measuring acceptance, appreciation, and understanding of the welfare of others (Schwartz, 2012). Semi-structured interviews can be used to capture rich, detailed nuanced answers that uncover subjective differences and specificities of the interviewee. Other interview methods we reviewed were either focused on self-transcendence as experiential, e.g., phenomenology or retrospective, or were about self-transcendence in-the-present-moment, e.g., microphenomenology, think-aloud -protocol, and cued recall debrief. Diary and journal methods may provide some context to why a participant may value self-transcendence or not.

**Developmental Process**

Reed (2013)'s theory of self-transcendence aligns with Frankl and Maslow's developmental stages that need to be fulfilled in order to have a sense of purpose in life. Reed's theory has been primarily used in older adult populations where self-transcendence plays an integral role in healing and in dignified acceptance of the end of life. From this theory, Reed et al. (1989) developed the Self-transcendence Scale (STS) to identify intrapersonal, interpersonal, transpersonal, and temporal experiences characteristic of later life, which reflect expanded boundaries of self. The Adult Self-Transcendence Inventory (ASTI) is another questionnaire to measure transcendence as a developmental process that is more lifespan inclusive (Levenson et al., 2005). Between the two, we recommend the STS because it is more widely used, it has higher internal consistency, and it reports good construct validity; whereas the ASTI has questionable test-retest reliability and construct validity.

We also recommend diary entries in this case because they obtain rich, longitudinal narratives of individuals' lives that can be a window into how self-transcendence develops over time. Retrospective interviews can also help to understand how self-transcendence as a developmental process changes over time.

**Personality Trait**

Personality traits are defined as descriptions of people in terms of relatively stable patterns of behavior, thoughts, and emotions, and are therefore summaries of an individual's responses and behaviors (McCrae and Costa, 2003). In order to assess self-transcendence as a personality trait, one should consider the following questionnaires that measure an individual's behavioral, cognitive, and emotional tendencies. The following all have good reliability and encompass both the broad and specific construct of self-transcendence, depending on what you are most interested in. Moreover, we found these questionnaires tended to span across different populations and faiths. That said, if one is interested in a specific context of self-transcendence, e.g., in the Christian faith or involving hallucinogenics, then it would be worth considering measures specifically with those in mind.

For general measures of self-transcendence as a trait, we recommend the Dispositional Positive Emotion Scales (DPES) that assess seven different positive emotions: joy, contentment, pride, love, compassion, amusement, and awe (Shiota et al., 2006). The second measure is Temperament and Character Inventory (TCI) (Cloninger et al., 1993b). In terms of measuring specific constructs of self-transcendence, the Mindful Attention Awareness Scale (MAAS) measures general tendency to be attentive to and aware of present moment experience in daily life (Brown and Ryan, 2003). Second, the Dispositional Flow Scale-2 (DFS-2) measures the frequency of flow experiences in chosen physical activity in general (Jackson and Eklund, 2002). Third, Gratitude/Awe Scale (GrAw-7) measures feelings of gratitude, reverence/awe, and experiencing the beauty in life (Büssing et al., 2018). Lastly, Spiritual Transcendence Scale (Spirit-TS) captures a personal tendency to turn toward a larger, objective perspective of reality than one's personal life (Piedmont, 1999).

When considering self-transcendence as a trait, rather than a state, the type of brain imaging used should reflect that goal. Self-transcendence as a state would require measuring brain activity, and thus would need a functional imaging method such as EEG, fMRI, PET, SPECT, or MEG. On the other hand, measuring self-transcendence as a trait can use either a functional imaging method such as EEG or a structural imaging method such as computerized tomography (CT) or MRI. As Cahn and Polich (2006) point out in their review paper on neuroimaging studies of meditation states and traits, EEG can be used to measure changes in brainwave amplitude in comparisons such as resting vs. meditating or beginner vs. expert meditators. In CT scanning, a computer is used to construct images of the brain (or other parts of the body) from a series of x-ray scans at multiple angles, giving much better resolution than standard x-rays. MRI scans, on the other hand, not only provide higher-definition images than CT scans, but they can also show sagittal and coronal sections of the brain, not just the axial sections to which CT scans are limited. While EEG measures coherence between brain activity or spectral values in different frequency ranges, MRI focuses on activations in different brain areas (Raffone and Srinivasan, 2010). Therefore, it appears the best way to measure self-transcendence as a trait is through a combined method of EEG and MRI, which several studies of meditation have already done (Cahn and Polich, 2006).

Since traits look at patterns of behavior, thoughts, and emotions, experience sampling can help to describe patterns of an individual's daily experience, to evaluate the common experience of situations, and to study the dynamics of emotions and other subjective states. Semi-structured interviews can also help determine patterns; although observation would directly measure behavior, it would be very time consuming to see stable patterns of behavior that are warranted for personality traits.

### 5.4. Future Directions

After reviewing the current measures of self-transcendence, we have recommended a multi-method approach with a list of different measures researchers should consider based on their validity, reliability, and suitability. We recommend this multi-method approach since self-transcendence is currently defined in many ways depending on the theoretical lens. As such, we divided our recommendations according to how one conceptualizes self-transcendence, namely as a state, trait, value, or developmental process. We recognize that we have recommended a long list of methods, and due to limited resources many researchers hoping to study self-transcendence may not have the means to use all of the measures we have recommended. In this case, we would still strongly suggest taking a multi-method approach and select the few measures that would be most relevant and accessible to the study. For example, questionnaires are a well-validated method of assessing some aspects of self-transcendence, and triangulating that data with a physiological measure and qualitative interviews can increase confidence in the findings. If one were to select only one type of measure, we might suggest taking a qualitative approach since at its core self-transcendence is a subjective phenomena that cannot be reduced to physiological and neurological correlates. Already, there are researchers taking a multi-method approach to measuring self-transcendence or one of its constructs and we might take these studies as examples of how to move forward in the field. Reinerman-Jones et al. (2013) used questionnaires, EEG, ECG, fNIRS, and phenomenological interviews to investigate awe and wonder in a simulated space travel scenario. Zhu et al. (2017) used a package of probes in the form of photos, diaries, and cards together with questionnaires, ECG, and semi-structured interviews to assess mindfulness.

One of the major challenges in measuring self-transcendence is in capturing it while it is happening. Recent developments in virtual reality (VR) have provided an opportunity to create realistic experiences that can support a self-transcendence. Thus, moving forward, we might use this technology as a way to further explore self-transcendence. Quesnel et al. (2018) used VR to help elicit awe and wonder during a journey through a forest, under water, and space to finally gaze upon the Earth. Quesnel and Riecke (2018) were able to elicit awe in four different Google Earth VR environments. Chirico et al. (2018) found that VR can effectively elicit feelings of awe, and that emotions elicited by virtual and natural conditions were not significantly different (Chirico and Gaggioli, 2019). With the development of VR headsets with integrated physiological and neurological sensors, it seems possible for VR to both help elicit self-transcendence and at the same time measure participant's bodily responses. Moreover, the use of first-person recordings of the virtual scene can be used for cued-recall debrief interviews.

Another challenge we identified in our review is that self-transcendence is measured differently depending on the theoretical lens used to define it. Self-transcendence as an emotional state seems to be the most widely used according to our review. One of the critiques of this view comes from embodied appraisal theory, which states that emotions are perceptions of bodily changes according to somatic theories of emotion (James, 1884). Here, emotions register changes in our bodies, but do not represent bodily changes (Prinz, 2004). Instead of response-dependent properties, emotions represent core relational themes (Lazarus and Lazarus, 1991), i.e., emotions have meaning. According to this theory, we can use physiological signals that correlate with self-transcendence, but self-transcendence, like any emotion, does not represent a change in body physiology. Self-transcendence does represent core relational themes, which inform us about our relationship with the world, embody our convictions, and factor intelligibly into our decisions. Although not everyone would agree with Prinz's embodied appraisal theory of emotions, e.g., Pineda (2015), future research on measuring self-transcendence should carefully consider what measures are actually correlations or proxies of self-transcendence rather than self-transcendence itself. Qualitative experience seems to be the key to measuring and understanding self-transcendence directly.

## 6. Conclusion

Within this paper, we described and reviewed measurement approaches to self-transcendence. Based on our review, we provided recommendations and suitability of methods given different research contexts. We want to reiterate that there is no one answer to the question “how does one measure self-transcendence.” The optimal choice needs to be determined by the researcher's objectives and the research questions themselves. We have seen that the construct of self-transcendence itself varies greatly depending on theoretical groundings. Whatever the theoretical framing, we suggest that researchers employ a mixed-methods approach in order to embrace the enactive quality of self-transcendence with empirical rigor.

## Author Contributions

AK, AG, and BR contributed to the initial planning and topic of the review. AC provided guidance on review conceptualization. AK performed the screening and eligibility process and conducted a qualitative research synthesis of the data. AC provided feedback and additional research synthesis. AK wrote the first draft of the manuscript. All authors contributed to manuscript revision, read, and approved the submitted version.

## Conflict of Interest

The authors declare that the research was conducted in the absence of any commercial or financial relationships that could be construed as a potential conflict of interest.
