# Corrigendum: A Review on Research and Evaluation Methods for Investigating Self-Transcendence

**DOI:** 10.3389/fpsyg.2021.682099

**Published:** 2021-04-19

**Authors:** Alexandra Kitson, Alice Chirico, Andrea Gaggioli, Bernhard E. Riecke

**Affiliations:** ^1^School of Interactive Arts and Technology, Simon Fraser University, Burnaby, BC, Canada; ^2^Department of Psychology, Catholic University of the Sacred Heart, Milan, Italy; ^3^ATN-P Lab, Istituto Auxologico Italiano, Milan, Italy

**Keywords:** self-transcendence, review, research methods, mindfulness, flow, peak experiences, neurophenomenolgy, emotional experiences

In the published article, there was an error regarding the affiliation(s) for Andrea Gaggioli. As well as having affiliation 2, she should also have affiliation 3, “ATN-P Lab, Istituto Auxologico Italiano, Milan, Italy.”

The authors apologize for this error and state that this does not change the scientific conclusions of the article in any way. The original article has been updated.

