# Supplementary material for: A Review on Research and Evaluation Methods for Investigating Self-Transcendence
Source: Front Psychol. 2020 Nov 16;11:547687. doi: 10.3389/fpsyg.2020.547687 (PMC7701337; doi:10.3389/fpsyg.2020.547687)
Supplement: Supplementary file 1 [file Data_Sheet_1.PDF]

Name	Reference	Goal	Basic Methods	Advantages	Disadvantages	Suitability	Reliability and Validity	Who's using this method?
**QUESTIONNAIRES AND SURVEYS**								
Inclusion of Other in Self Scale (IOS)	Aron, A., Aron, E. N., & Smollan, D. (1992)	Self/other overlap describes states of interpersonal connection	Participants select the picture that best describes their relationship from a set of 7 Venn-like diagrams each representing different degrees of overlap of two circles. The figures were designed so that (a) the total area of each figure is constant (thus as the overlap of the circles increases, so does the diameter), and (b) the degree of overlap progresses linearly, creating a seven-step, interval-level scale.	Easy to administer; Intuitive to Use; Takes less than one minute; Simple; Portable; Psychologically meanginful	Does not capture the subtle nuances and details of a relationship.	For measuring the closeness of relationships in intimate or aquaintance type relationships.	Alternate-form and test-retest reliability (r = .83 overall); convergent validity with the Relationship Closeness Inventory, Intimacy Scale, and other measures; discriminant validity; minimal social desirability correlations; and predictive validity for whether romantic relationships were intact 3 months later.	Van Cappellen and Saroglou (2012)
Dispositional Positive Emotion Scales (DPES)	Shiota, M. N., Keltner, D., & John, O. P. (2006)	To assess seven different positive emotions: joy, contentment, pride, love, compassion, amusement, and awe.	Participants report their level of agreement with each statement (39 items) on a 7-point scale anchored at (1) "strongly disagree" and (7) "strongly agree". Participants could also select −1 (refuse to answer).	Easy to administer; Does not take a lot of time; Portable	Does not assess positive emotions directly, but more people's dispositions toward positive emotions.	For positive emotion disposition, more related to personality traits.	Cronbach's alpha for each scale was: joy, 0.82; contentment, 0.92; pride, 0.80; love, 0.80; compassion, 0.80; amusement, 0.75; awe, 0.78. Construct validity: results of confirmatory factor analysis pointed that the model was acceptable fit.	Piff, P. K., Dietze, P., Feinberg, M., Stancato, D. M., & Keltner, D. (2015); Krenzer, W. L. (2018); Stellar, J. E., John-Henderson, N., Anderson, C. L., Gordon, A. M., McNeil, G. D., & Keltner, D. (2015); Stellar, J. E., Gordon, A., Anderson, C. L., Piff, P. K., McNeil, G. D., & Keltner, D. (2017); Guan, F., Chen, J., Chen, O., Liu, L., & Zha, Y. (2019); Chirico, A., & Gaggioli, A. (2019); Verhaeghen, P. (2019); Silvia, P. J., Fayn, K., Nusbaum, E. C., & Beaty, R. E. (2015)
Modified Differential Emotions Scale (mDES)	Fredrickson, B. L., Tugade, M. M., Waugh, C. E., & Larkin, G. R. (2003)	To assess individual's positive and negative emotions within a specific time frame, adding on eight additional positive emotions from the original DES.	Participants are asked to recall the past specified time (e.g., 24 hours) and rate their strongest experience of each of 20 specific emotions on a 5 point Likert scale (1—Not At All to 5—Extremely).	Easy to administer; Does not take a lot of time; Portable	Lack of consideration of low-energy states (e.g., fatigue and serenity); low internal consistency (reliability) due to few items.	Assesses discrete positive emotions amusement, awe, contentment, gratitude, hope, love, pride, sexual desire, joy, interest, and eight negative emotions plus surprise.	The Positive Emotions subscale is a composite of nine positive emotions (all but awe), with coefficient α= 0.79. The Negative Emotions subscale is a composite of 7 negative emotions (all but embarrassment), with coefficient α= 0.69. mDES can be used as a reliable tool for the assessment of positive and negative emotions in the Greek population. Specifically the Cronbach Alpha index for the scale was a= 0.751	Galanakis, M., Stalikas, A., Pezirkianidis, C., & Karakasidou, I. (2016); Deiters, J. (2016); Johnson, D. P., Penn, D. L., Fredrickson, B. L., Kring, A. M., Meyer, P. S., Catalino, L. I., & Brantley, M. (2011); Cann, A., & Collette, C. (2014); Schotanus-Dijkstra, M., Pieterse, M. E., Drossaert, C. H., Walburg, J. A., & Bohlmeijer, E. T. (2019); Nelson-Coffey, S. K., Ruberton, P. M., Chancellor, J., Cornick, J. E., Blascovich, J., & Lyubomirsky, S. (2019); Stell, A. J., & Farsides, T. (2016)
The Nature of Awe Questionnaire (NAQ)	Shiota, M. N., Keltner, D., & Mossman, A. (2007)	To document the self-diminishing effects of awe experience, and explore the effects of awe on the content of the self-concept.	Participants rate the appropriateness of the following words, on a scale from 1 (not appropriate at all) to 7 (very appropriate) for describing their emotions during the target experience: Awe, Contentment, Excitement, Fear, Joy, Love, Pride, Rapture, Sadness, and Surprise. Participants then rated how intense, personally meaningful, novel, challenging, and tiring the target experience was on a scale from 1 to 7. Finally, participants rated the extent to which they remembered having several appraisals of the target experience, using a scale from 1 (not true at all) to 7 (very true).	Easy to administer; Does not take a lot of time; Assesses intensity in addition to presence of emotion; Portable	Participants may have recall bias.	Assessing awe for a specific event.	These items tapped perceptions of vastness vis-à-vis the self and the accompanying sense of smallness, and they formed a reliable measure of the small self (α = .82).	Piff, P. K., Dietze, P., Feinberg, M., Stancato, D. M., & Keltner, D. (2015); Preston, J. L., & Shin, F. (2017); Stell, A. J. (2018)
Awe Experience Scale (AWE-S)	Yaden, D. B., Kaufman, S. B., Hyde, E., Chirico, A., Gaggioli, A., Zhang, J. W., & Keltner, D. (2018)	To assess a robust state measure of awe.	Participants answer 30 items regarding the single awe experience they had just described in writing. Each item was rated on a 7-point scale (1 = Strongly Disagree, 2 = Moderately Disagree, 3 = Somewhat Disagree, 4 = Neutral, 5 = Somewhat Agree, 6 = Moderately Agree, 7 = Strongly Agree).	Easy to administer; Portable	Internal consistency was strong for each factor(α≥.80)	This measure is intended for researchers interested in examining specific aspects of awe experiences.	The overall scale showed a high internal consistency (Cronbach Alpha = .92). AWE-S demonstrated adequate convergent, divergent, and construct validity when compared to similar questionnaires.	Graziosi, M., & Yaden, D. (2019); Yaden, D. B., Kaufman, S. B., Hyde, E., Chirico, A., Gaggioli, A., Zhang, J. W., & Keltner, D. (2019); Chirico, A., & Gaggioli, A. (2019); Nakayama, M., & Uchida, Y. (2019); Gabriel, S., Naidu, E., Paravati, E., Morrison, C. D., & Gainey, K. (2019); van Mulukom, V., Patterson, R., & van Elk, M. (2020)
Situational Awe Scale (SAS)	Krenzer, W. L. (2018)	To measure awe that reflects psychological, philosophical, and religious perspectives	Participants answer 18 items across four subscales on a Likert scale ranging from -3 (disagree strongly) to +3 (agree strongly).	Easy to administer; Portable; Can be used across multiple disciplines; Also measures "dark awe", or the more negative aspects associated with oppression/isolation.	Findings have not been replicated; Can miss the more nuanced and rich descriptions of awe experience.	SAS measures awe, correlates with the theoretically relevant constructs, and detects fluctuations in awe where other measures fail to do so.	Good structural, convergent, criterion, and discriminant validity. Internal consistency is good with alpha = .84 for total SAS and alpha ranges from .66 to .94 for interscale correlations.	Krogh-Jespersen, S., Quinn, K. A., Krenzer, W. L. D., Nguyen, C. T., Greenslit, J., & Price, A. (2020); Nakayama, M., & Uchida, Y. (2019); Gabriel, S., Naidu, E., Paravati, E., Morrison, C. D., & Gainey, K. (2019); Price, C. A., Quinn, K. A., Greenslit, J., Applebaum, L., Krogh-Jespersen, S., Krenzer, W. L. D., & Ibtasar, R. (2019); Krenzer, W. L. (2018)
Awe and the Small-self (AS)	Piff, P. K., Dietze, P., Feinberg, M., Stancato, D. M., & Keltner, D. (2015)	To measure the extent to which awe-inducing experiences influence perceived vastness and self-diminishment.	Participants report how much Anger, Anxiety, Awe, Disgust, Fear, Nervousness, Sadness, and Happiness they were feeling using single items (1 ┐ not at all, 7┐ extremely). Five additional items assess vastness vis-a`-vis the self; five items to assess self-diminishment.	Easy to administer; Portable	Validity unclear; Neglects the additional content suggested by treatments of awe conducted outside of psychology (e.g., admiration mixed with wonder and fear; sublime)	Assesses situational awe, mostly related to Psychological definitions of awe.	Cronbach's α = .92	Krenzer, W. L. (2018); Preston, J. L., & Shin, F. (2017); Chirico, A., Cipresso, P., Yaden, D. B., Biassoni, F., Riva, G., & Gaggioli, A. (2017); Chirico, A., Cipresso, P., Riva, G., & Gaggioli, A. (2016); Yang, Y., Hu, J., Jing, F., & Nguyen, B. (2018); Chirico, A., Glaveanu, V. P., Cipresso, P., Riva, G., & Gaggioli, A. (2018); Bai, Y., Maruskin, L. A., Chen, S., Gordon, A. M., Stellar, J. E., McNeil, G. D., ... & Keltner, D. (2017); Nelson-Coffey, S. K., Ruberton, P. M., Chancellor, J., Cornick, J. E., Blascovich, J., & Lyubomirsky, S. (2019)
Gratitude/Awe Scale (GrAw-7)	Büssing, A., Recchia, D. R., & Baumann, K. (2018)	To measure feelings of gratitude, reverence/awe, and experiencing the beauty in life.	Participants answer 7 questions on a four-point scale (0—never; 1—seldom; 2—often; 3—regularly).The resulting scores were sum scores ranging from 0 to 21.	Easy to administer; Portable; Short	The validation study does not assume that the study population is representative of a 'normal' population. Compared to life satisfaction and meditation, but not other awe scales. Relatively new, so not any other researchers using it at the moment.	Assesses gratitude/awe as a dispositional trait. This extended "Gratitude/Awe" questionnaire was suggested to represent states of mindful awareness rather than the direct reactions of gratefulness in response to the kindness of concrete persons.	Cronbach's alpha = 0.82; sound correlation with external measures, particularly with grateful disposition.	Büssing, A. (2019)
Flow State Scale (FSS)	Jackson, S. A., & Marsh, H. W. (1996)	To measure flow experienced within a particular event. Measures flow in sport and physical activity settings according to nine factors of flow: challenge-skill, action-awareness, clear goals, unambiguous feedback, concentration, sense of control, loss of self-consciousness, transformation of time, and autoletic experience.	Participants responded to the 36 flow items using a 5-point Likert-type response format (1 = strongly disagree to 5 = strongly agree).	Easy to administer; Portable; Takes little time to complete	Unclear whether nine factors should be weighted as a global score or not; generalizability outside sports unclear; does not provide rich descriptions of qualitative interviews	For sport and physical activity settings. Built on Csíkszentmihályi's theory of flow.	Internal consistency estimates for the nine FSS scales were reasonable (alpha M = 33) for administration of the scale to 394 athletes. Reliability α = .72 to .91	Morgan, J. D., & Coutts, R. A. (2016); Rufi, S., Wlodarczyk, A., Páez, D., & Javaloy, F. (2016); Monson, C. H. (2013); Kaufman, K. A., Glass, C. R., & Arnkoff, D. B. (2009)
Dispositional Flow Scale-2 (DFS-2)	Jackson, S. A., & Eklund, R. C. (2002)	To measure the frequency of flow experiences in chosen physical activity in general.	Participants respond to nine proposed components of flow. Each component is assessed with four items on a Likert scale, ranging from1 (never) to 5 (always).	Easy to administer; Portable; Takes little time to complete	It is dispositional rather than a state scale, so it measures the tendency to experience flow in a given setting rather than flow experience in a single given instance of activity.	It is a dispositional (or "trait") measure of flow that assesses how respondents generally feel when taking part in a given activity. Built on Csíkszentmihályi's theory of flow	Strong content and construct validity; reliability estimates range from .81 to .90.	Hamari, J., & Koivisto, J. (2014); Whitmore, J. G., & Borrie, W. T. (2006); Sinnamon, S., Moran, A., & O'Connell, M. (2012); Wang, C. J., Liu, W. C., & Khoo, A. (2009); Procci, K., Singer, A. R., Levy, K. R., & Bowers, C. (2012); Kawabata, M., Mallett, C. J., & Jackson, S. A. (2008)
Peak Scale (PS)	Mathes, E. W. (1982)	To objectively measure tendency to have peak experiences.	Participants respond to 70 true or false items, half of which are scored in the affirmative and half in the negative.	Easy to administer; Portable	Takes more time than most other questionnaires; Does not capture rich descriptions.	Tendencies for peak experience; not assessing peak experience themselves	Kuder-Richardson reliabilities for the scale are . 94 for males and .92 for females.	Christopher, J. C., Manaster, G. J., Campbell, R. L., & Weinfeld, M. B. (2002); Bassi, M., & Delle Fave, A. (2014); Macdonald (2000)

Name	Reference	Goal	Basic Methods	Advantages	Disadvantages	Suitability	Reliability and Validity	Who's using this method?
Experience Questionnaire (EQ)	Privette, G., & Bundrick, C. M. (1987)	To examine different experiences, such as peak experience, peak performance, misery, failure, sport, and average events.	Participants are first asked a stimulus question related to peak experience: "Will you describe one incident in your life characterized by highest happiness?" This is followed by 47 statements in Likert format and five scaled descriptions of the peak experience.	Easy to administer; Portable	Emphasizes inner retrospective experiences that may be prone to biases; Can take a long time to analyze phenomenological descriptions	Explore the phenomenon of peak experience	Test-retest reliability of the questionnaire was .70. Construct validity of experiential data has been supported by discriminant analysis, with a correct group classfication of 97.7 % of construct events.	Liang, Y. S. (2006); Lanier, L. S., Privette, G., Vodanovich, S., & Bundrick, C. M. (1996); Bassi, M., & Delle Fave, A. (2014); Verhaeghen, P. (2019)
Mysticism Scale (MS)	Hood, R. W., Jr. (1975)	To operationalize eight of the nine dimensions of mystical experience delineated by Stace (1960): Unifying Quality, Ineffability, Inner Subjective Quality, Temporal-Spatial Quality, Positive Affect, Religious Quality, Noetic Quality, and Ego Quality.	Participants rate their personal experiences on a 32 item self-report measure in which +2 (definitely true), +1 (probably true), -1 (probably not true), or -2 (definitely not true) depending on how participants feel in each case.	Easy to administer; Portable; One can study empirically the report of mystical experience	Does not measure the full range of feelings of realness associated with the noetic spectrum.	Investigation of mystical experiences, especially within the context of religious experience.	Sufficient construct validity and internal consistency (alpha = .93).	Monson (2012); Macdonald (2000); Griffiths, R. R., Johnson, M. W., Carducci, M. A., Umbricht, A., Richards, W. A., Richards, B. D., ... & Klinedinst, M. A. (2016); Yaden, D. B., Le Nguyen, K. D., Kern, M. L., Wintering, N. A., Eichstaedt, J. C., Schwartz, H. A., ... & Newberg, A. B. (2017); Skragge, M. (2017); Griffiths, R. R., Richards, W. A., Johnson, M. W., McCann, U. D., & Jesse, R. (2008); Garcia-Romeu, A., R Griffiths, R., & W Johnson, M. (2014); MacLean, K. A., Johnson, M. W., & Griffiths, R. R. (2011); Akyalcin, E., Greenway, P., & Milne, L. (2008)
Daily Spiritual Experience Scale (DSES)	Underwood, L. G., & Teresi, J. A. (2002)	To investigate ordinary spiritual experiences, such as awe, deep inner peace, giving and receiving compassionate love, and feeling thankful for blessings, and their role in daily life.	Participants rate 16 items on a 7-point Likert scale how often they directly have a given experience.	Easy to administer; portable; translated in many languages	The original sample was primarily Christian and later findings among a Jewish population indicate that norms may differ within other traditions.	Assesses subjective experiences (e.g., feelings and awareness) of interactions or involvement with the transcendent in daily life; used with adolescents and adults; mostly for religious or spiritual experiences.	High internal (alphas over .90) and test-retest reliability (Pearson r = .85 over two days), but factor analyses indicate that two items may load on a second factor. DSES has been used in more than 65 studies of psychological well-being. Used with adolescents and adults	Underwood, L.G. (2005); Wachholtz, A.B. and Pargament, K.I. (2005); Cole, B. S., Hopkins, C. M., Tisak, J., Steel, J. L., & Carr, B. I. (2008); Johnstone, B., Bodling, A., Cohen, D., Christ, S. E., & Wegrzyn, A. (2012); Greeson, J. M., Webber, D. M., Smoski, M. J., Brantley, J. G., Ekblad, A. G., Suarez, E. C., & Wolever, R. Q. (2011); Greeson, J. M., Smoski, M. J., Suarez, E. C., Brantley, J. G., Ekblad, A. G., Lynch, T. R., & Wolever, R. Q. (2015)
Spiritual Transcendence Scale (Spirit-TS)	Piedmont (1999)	To capture a personal tendency to turn towards a larger, objective perspective of reality than one's personal life.	Participants complete a 24-item questionnaire with item scoring ranging from 1(Strongly Disagree) to 5 (Strongly Agree).	Generalizes to a diverse range of faith traditions; Easy to administer; Portable;	Personality-based measure rather than as a phenomenon; Does not capture rich descriptions of spiritual transcendence	Assessing Spiritual Transcendence as a personality trait.	Satisfactory reliability (α= 0.86).	Skragge, M. (2017); Griffiths R. R., Richards W. A., McCann U., et al. (2006); Griffiths, R. R., Richards, W. A., Johnson, M. W., McCann, U. D., & Jesse, R. (2008); Saroglou, V., Buxant, C., & Tilquin, J. (2008); Walach H, Kaseberg E (1998); Kjellgren A, Sundequist U, Sundholm U, Norlander T, Archer T (2004); Akyalcin, E., Greenway, P., & Milne, L. (2008)
Mystical Experience Questionnaire (MEQ)	MacLean (2012)	To measure or characterize hallucinogen-occasioned spiritual experiences.	Participants answer a 30-item questionnaire rated on a six-point scale (0 = none, not at all; 1 = so slight cannot decide; 2 = slight; 3 = moderate; 4 = strong [equivalent in degree to any previous strong experience]; and 5 = extreme [more than ever before in my life and stronger than 4]). It takes approximately 30 minutes to complete.	Easy to administer; Portable; Generalizable to natural settings	Results may be biased toward positive spiritual experiences; Does not measure the full range of feelings of realness associated with the noetic spectrum.	Assesses the effects of hallucinogens in laboratory studies. The appropriateness of the scale for use in other populations or contexts is unclear.	MEQ factor scores showed good internal reliability (alpha = .933) and correlated with the Hood Mysticism Scale (r= .81, p < .001), indicating convergent validity. Participants who endorsed having had a mystical experience on psilocybin, compared to those who did not, had significantly higher factor scores (p < .001), indicating construct validity.	Griffiths, R. R., Johnson, M. W., Carducci, M. A., Umbricht, A., Richards, W. A., Richards, B. D., ... & Klinedinst, M. A. (2016); Barrett, F. S., Johnson, M. W., & Griffiths, R. R. (2015); Barrett, F. S., & Griffiths, R. R. (2017); Russ, S. L., & Elliott, M. S. (2017); Lynn, S. J., & Evans, J. (2017); Griffiths R. R., Richards W. A., McCann U., et al. (2006)
States of Consciousness Questionnaire (SOCQ)	Griffiths R. R., Richards W. A., McCann U., et al. (2006)	To assess mystical experiences on seven domains: internal unity (pure awareness; a merging with ultimate reality); external unity (unity of all things; all things are alive; all is one); transcendence of time and space; ineffability and paradoxicality (claim of difficulty in describing the experience in words); sense of sacredness (awe); noetic quality (claim of intuitive knowledge of ultimate reality); and deeply felt positive mood (joy, peace, and love).	Participants rate a 100-item questionnaire on a six-point scale [0=none, not at all; 1=so slight, cannot decide; 2=slight; 3=moderate; 4=strong (equivalent in degree to any previous strong experience or expectation of this description); and 5=extreme (more than ever before in my life and stronger than 4)]. 43 items came from the Mystical Experience Questionnaire; 57 items in the questionnaire served as distracter items.	Distractor items ensures whether a participant had a "complete" mystical experience or not; Easy to administer; Portable	Rather long questionnaire to complete	Similar to Mysticism Scale, expect more for hallucinogenic experiences.	The MEQ portion of questionnaire has shown good internal validity and reliability through exploratory and confirmatory statistical analysis. Cronbach's alpha: mystical (0.95); positive mood (0.71); transcendence of time/space (0.83); and ineffability (0.81).	Griffiths, R. R., Richards, W. A., Johnson, M. W., McCann, U. D., & Jesse, R. (2008); MacLean (2012); Liechti, M. E., Dolder, P. C., & Schmid, Y. (2017); Griffiths, R. R., Johnson, M. W., Carducci, M. A., Umbricht, A., Richards, W. A., Richards, B. D., ... & Klinedinst, M. A. (2016); Garcia-Romeu, A., R Griffiths, R., & W Johnson, M. (2014); MacLean, K. A., Johnson, M. W., & Griffiths, R. R. (2011)
Ego-Dissolution Inventory (EDI)	Nour, M. M., Evans, L., Nutt, D., & Carhart-Harris, R. L. (2016).	To measure the associated feeling of increased union with one's surroundings.	Participants answer 16 items rated using a visual analog scale format (0–100, with incremental units of one) with zero defined as "No, not more than usually", and 100 defined as "Yes, entirely or completely".	Short, easy to administer.	Retrospective measure; does not distinguish between alterations to different aspects of self-awareness; does not measure positive affect, ineffability, or transcendence of time and space	Measuring ego dissolution in terms of psychedelic expereinces.	Demonstrated convergent validity with the MEQ-derived measure of unitive experience (rho = 0.735). Limimted external validity due to homogenous sample. Internal consistency for ego-dissuolution (Cronbach's α =.81) and ego-inflation (Cronbach's α =.78)	Uthaug, M. V., Van Oorsouw, K., Kuypers, K. P. C., Van Boxtel, M., Broers, N. J., Mason, N. L., ... & Ramaekers, J. G. (2018); Timmermann, C., Roseman, L., Williams, L., Erritzoe, D., Martial, C., Cassol, H., ... & Carhart-Harris, R. (2018); Mason, N. L., Kuypers, K. P. C., Müller, F., Reckweg, J., Tse, D. H. Y., Toennes, S. W., ... & Ramaekers, J. G. (2020); Nour, M. M., Evans, L., & Carhart-Harris, R. L. (2017); van Mulukom, V., Patterson, R., & van Elk, M. (2020)
Nondual Awareness Dimensional Assessment-Trait (NADA-T)	Hanley, A. W., Nakamura, Y., & Garland, E. L. (2018).	To measure nondual awareness as a trait in terms of its two constructs: self-transcendence and bliss	Participants anser 13 questions on a five-point Likert scale: "Never or Very Rarely" to "Very Often or Always"	Easy to administer; portable; short	Does not capture the construct of "pristine awareness," ostensibly the "highest" form of nondual awareness. Quiered only positive experiences of nondual awareness.	Expressly measures the third and fourth modes of awareness: form and formless absorption (i.e., relational self-transcendence) and experiential emptiness of self (i.e., annihilational self-transcendence).	Composite reliability for the two dimensions was good [self-transcendence (.94) and bliss (.81)] and excellent for the full scale score (.93). The two dimensions were positively correlated (r=.62, p<.001).	Hanley, A. W., & Garland, E. L. (2019)
Nondual Awareness Dimensional Assessment-State (NADA-S)	Hanley, A. W., Nakamura, Y., & Garland, E. L. (2018).	To measure nondual awareness as a state.	Participants answer 13 questions on a 10-point Likert scale (1=Not at all, 10=Very Much)	Easy to administer; portable; short	Additional studies are needed to establish the convergent and discriminant validity of the NADA	The NADA-S was designed to be brief for assessing moment-to-moment changes in nondual states evoked during specific contemplative practices and during complex sequences of such practices (e.g., a mindful breathing practice, followed by a practice on the nature of self, followed by a nondual practice), examining the progressive deepening of nondual states over an extended meditation practice session (e.g., 60 minutes).	Items were taken from NADA-T. In a study with a brief mindfulness meditation session, this scale was able to detect changes in nondual awareness.	Hanley, A. W., & Garland, E. L. (2019)
Phenomenology of Consciousness Inventory (PCI)	Pekala, R. J. (1991)	To assess diverse aspects of conscious experience that range across 12 dimensions of consciousness.	Participants self-report 53-item questionnaire where items are scaled from 0 (e.g., the absence of a phenomenon) to 6 (e.g., continued or strong presence of the phenomenon).	Easy to administer; Portable	Does not capture the rich details of individual experience	Systematic, quantitative assessment not only of the different putative dimension or structures of consciousness, but also of states, including altered states, of consciousness.	Internal consistency for the PCI as alpha=.82 for all major dimensions combined, and .75 to .82 for the awakened experience (AE) domain. PCI is reported to have adequate reliability, as well as construct, discriminant, and predictive validity.	Roussel, J. R., & Bachelor, A. (2000); Maitz, E. A., & Pekala, R. J. (1991); Marcusson-Clavertz, D., & Cardeña, E. (2011)

Name	Reference	Goal	Basic Methods	Advantages	Disadvantages	Suitability	Reliability and Validity	Who's using this method?
Altered States of Consciousness Rating Scale (OAV)	Bodmer, I., Dittrich, A., & Lamparter, D. (1994); Studerus, E., Gamma, A., & Vollenweider, F. X. (2010)	To measure positively experienced depersonalization and derealization, deeply-felt positive mood, and experiences of unity.	Participants answer a 66-item yes/no questionnaire to the described experiences by placing marks on horizontal visual analogue scales (VAS) of 100 millimeters length. VAS was anchored as no, not more than usual on the left and as yes, very much more than usual on the right	Shorter version of 5D-ASC; Easy to administer	A study examining the factorial structure of OAV showed the original three dimensional structure was not supported; only assesses high level of the construct.	For self-report assessment of subjective experiences of ASC in retrospect. The original scale is not validated, and a recent study showed the new scales of OVA may provide a better fit and lower order scales do have sufficient validity and reliability (42-items).	Cronbach's alpha for the 66-item original scale is .96. New items have alphas ranging from .73 - .91	Studerus, E., Gamma, A., & Vollenweider, F. X. (2010)
5-Dimension Altered States of Consciousness (5D-ASC)	Dittrich (1998) & Dittrich, A., Lamparter, D., & Maurer, M. (2010)	To assess altered states of consciousness according to five dimensions: oceanic boundlessness, dread of ego dissolution, visonary restructuralization, auditory alterations, and vigilance reduction.	Participants answer either a 72-item yes/no questionnaire (1998) or a 94-item questionnaire (2010) to the described experiences by placing marks on horizontal visual analogue scales (VAS) of 100 millimeters length. VAS was anchored as no, not more than usual on the left and as yes, very much more than usual on the right	Has been translated into many different languages and applied internationally in approximately 70 experimental studies.	Binary measure may be too crude to measure subtle alterations of consciousness; A study found the originally proposed model did not fit the data well (Studerus et al., 2010).	For assessing subjective experiences of altered states of consciousness in retrospect.	Reliabilities and validities of this scale was deemed to be acceptable in the experimental as well as in the field studies Cronbach's alpha = .93	Liechti, M. E., Dolder, P. C., & Schmid, Y. (2017); Griffiths, R. R., Johnson, M. W., Carducci, M. A., Umbricht, A., Richards, W. A., Richards, B. D., ... & Klinedinst, M. A. (2016); Griffiths R. R., Richards W. A., McCann U., et al. (2006); MacLean, K. A., Johnson, M. W., & Griffiths, R. R. (2011); Studerus, E., Gamma, A., Kometer, M., & Vollenweider, F. X. (2012)
11-Dimension Altered States of Consciousness (11D-ASC)	Studerus, E., Gamma, A., and Vollenweider, F. X. (2010)	To assess altered states of consciousness according to 11 dimensions: experience of unity, spiritual experience, blissful state, insightfulness, disembodiment, impaired control and cognition, anxiety, complex imagery, elementary imagery, audio-visual synesthesia, and changed meaning of percepts.	Participants answer a 94-item questionnaire, each item is scored as in a visual analogue scale with the upper anchor reading “much more than usual” and the bottom one reading “no more than usual”.	Meets criteria of unidimensionality and therefore are more homogeneous than the 5D-ASC; well fitting basic measurement model; differentiated better among the three drug groups than the 5D-ASC	Slightly less reliable compared to 5D-ASC	Self-rating scale of psychological experiences of psilocybin can be measured across drug conditions, settings, and sexes.	Using Cronbach's alpha, only two of the 11 scales (insightfulness and spiritual experience) had reliabilities smaller than 0.8. However, both of these scales consisted of only three items and their reliabilities were still above 0.7, which indicates modest reliability. Correlations between other scales supported the convergent and discriminant validities.	Carhart-Harris, R. L., Bolstridge, M., Rucker, J., Day, C. M., Erritzoe, D., Kaelen, M., ... & Taylor, D. (2016); Carhart-Harris, R. L., Bolstridge, M., Day, C. M. J., Rucker, J., Watts, R., Erritzoe, D. E., ... & Rickard, J. A. (2018); Kometer, M., Pokorny, T., Seifritz, E., & Volleinweider, F. X. (2015)
Self-transcendence Scale (STS)	Reed, P., Boyd, M., & Buckwalter, K. (1989)	To identify intrapersonal, interpersonal, transpersonal, and temporal experiences characteristic of later life, which reflect expanded boundaries of self.	Participants answer 15 items on a scale from 1 (not at all) to 4 (very much). The possible range of scores is 15–60, with 60 representing the highest degree of self-transcendence.	Measures a central dimension correlated to well-being; Used in many studies in nursing; Portable; Easy to administer	Measures self-transcendence as a trait only and not a state; Missing subtle nuances of qualitative experience.	Items were developed to avoid bias toward the physically able or healthy person and rather measure the person's ability to derive a sense of well-being through cognitive, creative, social, spiritual, and introspective resources. The STS has been adapted for use with adolescent, adult, and older adult populations.	Reliability has been calculated by Cronbach's alpha, which ranged from .80 to .88, with test–retest reliability of .95. Content (compared to literature) and construct (measured against other scales) validity adequate.	Coward, D. D. (1991); Coward, D. D. (1996); Ellermann, C. R., & Reed, P. G. (2001); Kirk, K. M., Eaves, L. J., & Martin, N. G. (1999); McCarthy, V. L., Hall, L. A., Crawford, T. N., & Connelly, J. (2018); Haugan, G., Rannestad, T., Hammervold, R., Garåsen, H., & Espnes, G. A. (2014); Kang, Y., & Son, H. (2018); Garcia-Romeu, A. P. (2012); Runquist, J., & Reed, P. (2007)
Self Expansiveness Level Form (SELF)	Friedman (1983)	To measure self-concept expansiveness which assesses individual differences in self-concept in terms of temporality and space.	Participants answer 18. questions on a 5-pt Likert scale. Participants are asked to choose the letter which best expresses their willingness to use that concept as an answer to the question, "WHO AM I?". A = very willing to use to describe my sense of self for identity; B = somewhat willing; C = neither willing nor unwilling; D = somewhat unwilling; E = very unwilling to use to describe my sense of self or identity.	Measures ego disillusionment and attempts to qualitify self-experience.	Individuals may also portray themselves as highly transpersonal/spiritual out of “spiritual desirability” (e.g., for egoic reasons or a type of spiritual posturing)	SELF is designed to assess self-expansiveness, which has been operationalized as three distinct levels based on a spatial-temporal cartography of self-concept: personal, middle, and transpersonal.	Cronbach's alpha = .66-.81; Test-retest reliability r = .8-.83. In terms of convergent validity, the transpersonal subscale has been significantly correlated to the Mystical Experiences Scale (Hood, 1975). The personal subscale has been significantly correlated to the Time Competence Scale and the Inner Supports Scale of the Personal Orientation Inventory (Shostrom, 1963), and the Tennessee Self Concept Scale (Fitts,1965) Total Positive Score and the Self-Description Inventory Total Score. Criterion validity has also been shown to be satisfactory as was indicated by significant correlations found between personal and transpersonal subscale scores and a yoga teacher's ratings of transpersonal realization found in a group of yoga students .	MacDonald, D. A., Tsagarakis, C. I., & Holland, C. J. (1994); Pappas, J. D., & Friedman, H. L. (2007)
Temperament and Character Inventory (TCI)	Cloninger, C. R., Svrakic, D. M., & Przybeck, T. R. (1993)	To measure self-transcendence as a trait, which refers generally to "identification with everything conceived assessential and consequential parts of a unified whole".	Participants complete a self-report questionnaire according to seven subscales, one of which is self-transcendence. The original TCI was comprised of 226 items with true/False answers. The revised TCI has 240 items using a 5-point Likert scale. The self-transcendence portion of the questionnaire is comprised of 33 True/False items (TCI) or 26 5-point Likert scale items (TCI-R).	Easy to administer; Portable; small number of items with good reliability and validity; TCI widely used in over 400 studies	Ignores developmental process of self-transcendence; Not inclusive of religious/spiritual components of self-transcendence	Psychometrically sophisticated measure of transcendence that has been administered for research purposes in a variety of languages and disciplines.	High internal consistency for both TCI (alpha = .84) and TCI-R (alpha = .90); satisfactory reliability and adequate convergent and criterion validity.	Akyalcin, E., Greenway, P., & Milne, L. (2008); Pelissolo, A., Mallet, L., Baleyte, J. M., Michel, G., Cloninger, C. R., Allilaire, J. F., & Jouvent, R. (2005); MacDonald, D. A., & Holland, D. (2002); Comings, D. E., Gonzales, N., Saucier, G., Johnson, P. J., & MacMurray, J. P. (2000); Kirk, K. M., Eaves, L. J., & Martin, N. G. (1999); Matiz, A., Fabbro, F., & Crescentini, C. (2018); Garcia-Romeu, A. P. (2012); Borg, J., Andrée, B., Soderstrom, H., & Farde, L. (2003); Hakamata, Y., Iwase, M., Kato, T., Senda, K., & Inada, T. (2013); Karlsson, H., Hirvonen, J., Salminen, J. K., & Hietala, J. (2011); Fiori, F., David, N., & Aglioti, S. M. (2014)
Adult Self-Transcendence Inventory (ASTI)	Levenson, M. R., Jennings, P. A., Aldwin, C. M., & Shiraishi, R. W. (2005)	To measure transcendence as a developmental process that is more lifespan inclusive.	Participants answer 18 (34 for the revised version, where 10 are alienation items) Likert-scaled items ranging from 1 (disagree strongly) to 4 (agree strongly) based on the last five-ten years of their life.	Easy to administer; Portable; Distractor items support greater validity	Some subdimensions represent important preconditions, correlates, or even outcomes of self-transcendence instead of the experience of self-transcendence itself; large variability between participants	Mostly used to assess wisdom because self-transcendence is considered a componet of wisdom; It's recommeneded to use the subdimension scores rather than the overall score for more accurate reflection of self-transcedence measurement.	Its reliability is satisfactory (α =.83), and moderate internal consistency of .66 for the ASTI self-transcendence scale. The original ASTI has demonstrated high levels of face validity, moderate convergent validity, and moderate-to-high levels of internal reliability.	Ardelt, M., & Oh, H. (2015); Koller, I., Levenson, M. R., & Glück, J. (2017); Camkiran, S. (2018); Garcia-Romeu, A. P. (2012); Beaumont, S. L. (2009); Abdollahi, A., Panahipour, H., Allen, K. A., & Hosseinian, S. (2019)
Portrait Values Questionnaire Revised-RR-Self-transcendence Subscale (PVQ-RR-ST)	Schwartz (2012)	To measure self-transcendence as a value as it relates to values of universalism and belevolence, specifically measuring acceptance, appreciation, and understanding of the welfare of others.	Participants answer 15 items on a 6-pt scale from 1 = not like me at all to 6 = very much like me.	Easy to administer; Portable	Values are not the same as traits, so need to be careful conflating the two.	PVQ is an alternative to the Schwartz Value Survey (SVS) that measures the ten basic values of persons not educated in Western schools that emphasize abstract, context-free thinking. It can be used with children as well as adult populations.	Internal consistency ranges from 0.76 to 0.85.	Florez, I. A., Schulenberg, S. E., Lair, E. C., Wilson, K. G., & Johnson, K. A. (2019)
Mindful Attention Awareness Scale (MAAS)	Brown, K. W., & Ryan, R. M. (2003)	To measure general tendency to be attentive to and aware of present moment experience in daily life.	Participants answer 15-item 6-pt Likert scale questionnaire	Most widely used measure of dispositional mindfulness; Easy to administer; Short time to complete; Portable	Does not have sub-scales, so difficult to analyze self-transcendent qualities of mindfulness; Most studies use college, white males as the sample, so generalizability is not high	Measures mindfulness as a trait that involves two components of consciousness: awareness and attention.	Internal consistency of .82 and expected convergent and discriminant validity correlations. Test-retest reliability is 0.81; Adequate convergent and discriminant validity. The construct validity of Spanish version of mindfulness is concurrent with the findings of the English version and Cronbach's alpha reliability coefficient was 0.89.	Gervais, R., Roo, J. S., Frey, J., & Hachet, M. (2016); Navarro-Haro, M. V., López-del-Hoyo, Y., Campos, D., Linehan, M. M., Hoffman, H. G., García- Palacios, A., ... García-Campayo, J. (2017); Birnie, K., Speca, M., & Carlson, L. E. (2010); Stewart, M., & Haaga, D. A. (2018); Panno, A., Giacomantonio, M., Carrus, G., Maricchiolo, F., Pirchio, S., & Mannetti, L. (2017); Matiz, A., Fabbro, F., & Crescentini, C. (2018); Beitel, M., Bogus, S., Hutz, A., Green, D., Cecero, J. J., & Barry, D. T. (2014); Jislin-Goldberg, T., Tanay, G., & Bernstein, A. (2012); Carmody, J., Reed, G., Kristeller, J., & Merriam, P. (2008)

Name	Reference	Goal	Basic Methods	Advantages	Disadvantages	Suitability	Reliability and Validity	Who's using this method?
Freiburg Mindfulness Inventory (FMI)	Buchheld, N., Grossman, P., & Walach, H. (2001)	To measure non-judgemental present moment observation and openness to negative experience.	Participants answer a 30-item 4-pt Likert scale questionnaire	Easy to administer; Short time to complete; Portable; Heavily qualitative rather than quantitative measure	Cannot split into subscales; Because some familiarity with mindfulness practices is required when responding to this assessment, the meaning of some items can be unclear to persons without meditation experience	Measures trait mindfulness, with curiosity as an important piece of mindfulness.	Internal consistencies of .93 and .94.	Ovington, L. A., Saliba, A. J., & Goldring, J. (2018); Matiz, A., Fabbro, F., & Crescentini, C. (2018); Gaiswinkler, L., & Unterrainer, H. F. (2016); Mathad, M. D., Rajesh, S. K., & Pradhan, B. (2017); Hamann, G. A., & Ivtzan, I. (2016); Walach, H., Buchheld, N., Buttenmüller, V., Kleinknecht, N., & Schmidt, S. (2006)
Kentucky Inventory of Mindfulness Skills (KIMS)	Baer, R. A., Smith, G. T., & Allen, K. B. (2004)	To measure observation, describing, acting with awareness, and accepting without judgment.	Participants answer a 39-item 5-pt Likert scale questionnaire	Easy to administer; Short time to complete; Portable	Based on the conceptualization of mindfulness from a specific type of psychotherapy; Contains multiple subscales that provide redundant information	A scale that even casual meditators and irregular practitioners of mindfulness would be able to complete with ease. Measure trait mindflness	Internal consistencies range from .76 to .91 for the four sub-scales.	Beitel, M., Bogus, S., Hutz, A., Green, D., Cecero, J. J., & Barry, D. T. (2014); Kaufman, K. A., Glass, C. R., & Arnkoff, D. B. (2009)
Cognitive and Affective Mindfulness Scale Revised (CAMS-R)	Feldman, G., Hayes, A., Kumar, S., Greeson, J., & Laurenceau, J. P. (2007)	To measure attention, awareness, present-focus, and acceptance/nonjudgment with respect to thoughts and feelings in general daily experience.	Participants answer a 12-item 4-pt Likert scale questionnaire	Shortest holistic mindfulness measure; Easy to administer; Short time to complete; Portable	Does not have multiple sub-scales, so difficult to analyze self-transcendent qualities of mindfulness	Measure of trait mindfulness; Effectively captures a multi-component measurement of mindfulness and can be relied upon to relate with other measures as expected.	Internal consistencies were .74 to .80.	Matiz, A., Fabbro, F., & Crescentini, C. (2018); Greeson, J. M., Webber, D. M., Smoski, M. J., Brantley, J. G., Ekblad, A. G., Suarez, E. C., & Wolever, R. Q. (2011); Greeson, J. M., Smoski, M. J., Suarez, E. C., Brantley, J. G., Ekblad, A. G., Lynch, T. R., & Wolever, R. Q. (2015)
Southampton Mindfulness Questionnaire (SMQ)	Chadwick, P., Hember, M., Symes, J., Peters, E., Kuipers, E., & Dagnan, D. (2008)	To measure a mindful approach to distressing thoughts and images.	Participants answer a 16-item 7-pt Likert scale questionnaire	Easy to administer; Short time to complete; Portable	It is difficult to assign a score to a trait that is based on the ability to enter a state; Not widely used	Measure of trait mindfulness with the related constructs of openness to experience, emotional intelligence, and self-compassion.	Internal consistency alpha = .89; significant correlation with MAAS r= .57.	Keng, S. L., Robins, C. J., Smoski, M. J., Dagenbach, J., & Leary, M. R. (2013); Chadwick, P., Hughes, S., Russell, D., Russell, I., & Dagnan, D. (2009)
Five-Facet Mindfulness Scale (FFMQ)	Baer, R. A., Smith, G. T., Hopkins, J., Krietemeyer, J., & Toney, L. (2006)	To explore five facets relating to mindfulness: observing, describing, acting with awareness, non-judging of inner experience, and non-reactivity to inner experience.	Participants answer 39 items and uses a 5-pt Likert scale	Easy to administer; Short time to complete; Portable	Includes redundant subscales; Relatively long; Time consuming	Measures mindfulness as a trait that is correlated with the related constructs of openness to experience, emotional intelligence, and self-compassion	The five sub-factors of mindfulness showed strong internal consistency (Cronbach`s alpha >.70).	Gervais, R., Roo, J. S., Frey, J., & Hachet, M. (2016); Matiz, A., Fabbro, F., & Crescentini, C. (2018); Hanley, A. W., Derringer, S. A., & Hanley, R. T. (2017); Hanley, A. W., Warner, A., & Garland, E. L. (2015); Hanley, A. W., Baker, A. K., & Garland, E. L. (2017); Verhaeghen, P. (2019)
Meditation Depth Questionnaire (MEDEQ)	Piron, H. (2001)	To measure meditation depth along five developmental stages five stages of increasing depth.	Participants answer 30 items using a 5-pt Likert scale ranging from 0 (not at all) to 4 (very much) according to meditation experiences in the past week.	Easy to administer; Short time to complete; Portable	The creator mentions MEDEQ probably doesn't measure enlightnment, but is a good step toward a better measure of it.	Works across different types of meditation since it was tested with 40 expert meditators with different practices and found similar scores.	Convergent validity (.64-.93) with Meditation Development Index (Engel 1997); Spearman-Brown reliability = .90; divergent reliability with the Personality Inventory called TPF and the Symptom Check List. Internal consistency (Cronbach's alpha = 0.92).	Gobec, S., & Travis, F. (2018); Kosunen, I., Salminen, M., Järvelä, S., Ruonala, A., Ravaja, N., & Jacucci, G. (2016, March); Johnson, M. (2011); Ireland, M. J. (2013); Costa, M. R., Bergen-Cico, D., Grant, T., Herrero, R., Navarro, J., Razza, R., & Wang, Q. (2019)
State Mindfulness Scale (SMS)	Tanay, G., & Bernstein, A. (2013)	To capture state mindfulness instead of trait mindfulness	Participants answer 23 items and uses a 5-pt Likert scale	Easy to administer; Short time to complete; Portable	Retrospective bias; You can't assess mindfulness state while the participant is actively practicing mindfulness; Not widely used	Beneficial if you would like to measure your level of mindfulness in a particular recent mindfulness session.	Demonstrated high reliability, Cronehbach's alpha = .95	Stewart, M., & Haaga, D. A. (2018); Jislin-Goldberg, T., Tanay, G., & Bernstein, A. (2012); Sliwinski, J., Katsikitis, M., & Jones, C. M. (2018)
Toronto Mindfulness Scale (TMS)	Lau, M. A., Bishop, S. R., Segal, Z. V., Buis, T., Anderson, N. D., Carlson, L., ... & Devins, G. (2006)	To measure mindfulness in those actively practicing it.	Participants answer 13 items and uses a 5-pt Likert scale; comprised of two subscales: decentering and curiosity	Easy to administer; Short time to complete; Portable	Intended for meditators, so language used may not be accessible to novices.	This scale may be most helpful to those who would like to get an idea of their mindfulness level during a recent mindfulness practice.	Internal consistency curiosity alpha = .93; decentering alpha = .91; Demonstrated discriminant validity	Kitson, A., Riecke, B. E., & Vidyarthi, J. (2014); Kaufman, K. A., Glass, C. R., & Arnkoff, D. B. (2009); Carmody, J., Reed, G., Kristeller, J., & Merriam, P. (2008)
**DIARY AND JOURNAL ENTRY**								
Diary Entries	-	To obtain longitudinal narratives of individuals' lives.	Participants write accounts of important or particular events, e.g., dreams.	Can obtain new information about phenomenon; Can integrate into other models	Not easily quantifiable; Harder to integrate into existing models; Can be very selective and not representative of the whole experience; Events can be filtered or embellished	Suitable for studying the long-term effect of an event; when the event cannot be studied in a laboratory condition; when researchers want to know how participants reflect about an experience through time	One-week diaries have satisfactory construct validity (convergent, predictive and nomological); and, for salient activities, they are less subjective to retrospective bias than are interview data.	Cangas, A. J., Sass, L. A., & Pérez-Álvarez, m. (2008); Hall, C. S., & Van de Castle, R. L. (1966); Sokel, W. H. (1978); Thrash, T. M., & Elliot, A. J. (2003); Thrash, T. M., Elliot, A. J., Maruskin, L. A., & Cassidy, S. E. (2010); Zhu, B., Hedman, A., Feng, S., Li, H., & Osika, W. (2017).
Narrative Recall	Griskevicius, V., Shiota, M. N., & Neufeld, S. L. (2010)	Induced specific emotions by having participants recall and write about a personal experience involving the theoretically defined, prototypical elicitor of each emotion.	Participants recall and write about a personal experience involving the theoretically defined, prototypical elicitor of each emotion. Do not ask participants to write about "emotion word". Instructions should not include the target emotion word or any other emotion word.	Definition not constrained by researchers; Participants can give rich descriptions of positive emotions; Easy to administer	Time consuming to analyze	Eliciting and analyzing specific positive emotions.	Has been used in emotion research for decades as a well-validated measure.	Shaver, P., Schwartz, J., Kirson, D., & O'connor, C. (1987); Tangney, J. P., Miller, R. S., Flicker, L., & Barlow, D. H. (1996); Shiota, M. N., Keltner, D., & Mossman, A. (2007); Piff, P. K., Dietze, P., Feinberg, M., Stancato, D. M., & Keltner, D. (2015); Zhao, H., Zhang, H., Xu, Y., Lu, J., & He, W. (2018); Yang, Y., Hu, J., Jing, F., & Nguyen, B. (2018).
Experience Sampling	Csikszentmihalyi, M., Larson, R., & Prescott, S. (1977)	To provide a valid instrument to describe variations in self-reports of mental processes.	Participants report (e.g., write notes or type in an app) what they were experiencing just before the prompt of a pager or similar signaling device. The signals are typically given at random times to minimize instrumentation effects based on the expectation of a page at a particular time.	Can be easily used outside of the laboratory, thus enhancing its ecological validity. Because it includes repeated measures, it is also likely to provide a more representative view of the event investigated than single-testing techniques; obtains reports about people's experiences as they occur, thereby minimizing the effects of reliance on memory and reconstruction.	Does not give an indication of how transient experiences may impact or be integrated into the participant's ongoing or long-term narrative; dependence on respondents' self-reports; intrusive to participants	To describe patterns of an individual's daily experience; to evaluate the common experience of situations; to study the dynamics of emotions and other subjective states	Researchers recommend using other measures to corroborate the results and ensure validity. Acceptable levels of internal consistency and test-retest reliability	Gaggioli, A. (2012); Easterlin, B. L., & Cardeña, E. (1998); Smallwood, J., & Schooler, J. W. (2006); Jackson, S. A. (2000); Panter, A. J. (2017); Abuhamdeh, S., & Csikszentmihalyi, M. (2012); Graham, J. M. (2008); Fullagar, C. J., & Kelloway, E. K. (2009).
**INTERVIEWS**								
Phenomenolgical Interview	Bevan, M. (2014); Colaizzi, P. (1978)	To tease out the individual's perceptions of the experience	Participants recount their experiences with questions should be broad and open ended so the participant can express their view point extensively and limit interviewer bias	Thick, rich descriptions; Essense of experience or phenomenon emerges from participants rather than existing theory or research; Does not impose existing theoretical models; Comprehensive	May be too structured for some researchers; Understanding of assumptions required; Philosophical ideas are abstract; Participants need to be carefully chosen; Finding participants may be difficult; Bracketing personal experiences needed; Time consuming	Describing the lived experiences relating to self-transcendence.	Interviewing guidelines bring consistency and realiability to an otherwise very open-ended approach.	Panzarella, R. (1980); Kitson, A., Schiphorst, T., and Riecke, B. E. (2018); Gallagher, S., Janz, B., Reinerman, L., Trempler, J., & Bockelman, P. (2015); Kjellgren, A., Lyden, F., & Norlander, T. (2008); Downey, L. L. (2015); Coward, D. (1990); Coward, D. D., & Lewis, F. M. (1993); Coward, D., & Kahn, D. (2005);

Name	Reference	Goal	Basic Methods	Advantages	Disadvantages	Suitability	Reliability and Validity	Who's using this method?
Micro-phenomenology Interviews	Petitmengin C. (2006)	To obtain explicit descriptions of singular experiences as they unfold in a larger, chronological structure of the experience, bypassing generalized typical post-hoc descriptions by getting participants into an evocative state.	Researchers guide the interviewed persons from a general description towards the description of a singular experience, that is situated in space and time, and help them to mobilize their concrete memory to evoke this experience.	Rigourous technique; high level of reliability; fine degree of granularity; Comprehensive	Need highly trained interviewer; Time intensive	Explore lived experience very finely; singular event.	Rigourous technique and interviewer must comply with the rules during the interview;	Quesnel, D., Stepanova, E. R., Aguilar, I., Pennefather, P., and Riecke, B. E. (2018); Petitmengin, C., Van Beek, M., Bitbol, M., & Nissou, J. M. (2017); Prpa, M., Tatar, K., Françoise, J., Riecke, B., Schiphorst, T., & Pasquier, P. (2018); Stepanova, E. R., Quesnel, D., & Riecke, B. E. (2019); Timmermann, C., Roseman, L., Schartner, M., Milliere, R., Williams, L., Erritzoe, D., ... & Turton, S. (2019); Przyrembel, M., & Singer, T. (2018).
Thinking Out Loud	Watson, J. (1920)	To obtain ongoing verbal reports of the contents of consciousness.	Participants verbally report what they are experiencing while they are experiencing it.	Can obtain new information about phenomenon; Can integrate into other models; No participant bias that come from retrospective reports	Not easily quantifiable; May not be conprehensive account; Chance of reactivity during the process; Some experiences may not translate well because of temporal and representational limitations of language and translation between thought and experience;	Useful when a conprehensive ongoing account of an experience is desired.	Good reliability; invalidity due to problems with working memory	Marcusson-Clavertz, D., & Cardeña, E. (2011); Paterson, B., Thorne, S., Crawford, J., & Tarko, M. (1999); Aslan, I., Burkhardt, H., Kraus, J., & André, E. (2016)
Cued-Recall Debrief (CRD)	Omodei, M., & Mclennan, J. (1994)	To elicit information about participants' affect during system use.	Participants are recorded in the first-person perspective while engaging in an experience. A first-person recording is then immediately shown to the participant and they are encouraged to voice their thoughts and feelings during this process. Physiological signals are also recorded upon viewing the recording. The facilitator notes any reactions and may query the participant or pause the video. The comments are then critically examined to construct an overall view of the participant's thoughts, emotions, and affect, what influences were acting upon these, and how this impacted behaviour and choices.	Non-intrusive or disruptive of the experience while it happens; Can gather rich data; Significantly reduces the memory bias	One limitation is the proficiency of the facilitator's interviewing skills as well as the participant's willingness and comfort in sharing their feelings. Another limitation is using physiological signals for the CRD only; the CRD session may not elicit the same thoughts, emotions, and affect as originally experienced.	For studying experience in a natural setting without be intrusive or disruptive. Recommended in a number of applications including the environments of emergency services, therapeutic contexts, education and training, and sports psychology. Used for understanding the emotional and affective components of an experience as well as the performance and usability.	High inter-rater reliability (R=0.9831); Good concurrent validity	Bentley, T., Johnston, L., & von Baggo, K. (2005); Omodei, M. M., Wearing, A. J., and McLennan, J. (1998); Bruun, A., Law, E. L. C., Heintz, M., & Eriksen, P. S. (2016); Bruun, A., & Ahm, S. (2015); Quesnel, D., Stepanova, E., Aguilar, I., Pennefather, P., Riecke, B. E. (2018) ; Stepanova, E. R., Quesnel, D., & Riecke, B. E. (2019)
Retrospective Reports	Maslow, A. (1959)	To tap the various components of peak experiences.	Participants are asked to describe the most wonderful or joyful experience of their lives, how they felt, and if and how it affected their lives through in-depth interviews or open-ended surveys (15-30 minutes).	Usually only two questions; Rich descriptions; Does not require a lot of time to administer; Has been translated into multiple langauges	Lots of time to analyze results; Participant retrospective bias	Explore peak experiences	Coding scheme has been developed. Use member checking for improved validity.	Hoffman, E., & Muramoto, S. (2007); Hoffman, Iversen, & Ortiz. (2010); Bassi, M., & Delle Fave, A. (2014)
Semi-structured	Brinkmann, S., & Kvale, S. (2015)	To capture rich, detailed nuanced answers that uncover subjective differences and specificities of the interviewee.	Participants verbally respond to questions that were created beforehand. Normally all of the questions are asked, and similar wording is used from interviewee to interviewee. However, sometimes questions may not always follow the exact order in.	It allows more freedom to modify and add research ideas once the investigation has begun; includes greater interest in the interviewee's perspectives and concerns; encourages divergent interviewing in order to provide new insight based on relevant and important interviewee perspectives; allows flexible responding to the direction of the interviewee's responses enabling emerging issues to evolve.	Time consuming to collect and analyze; good data depends on the effetiveness of the interviewer	If the researcher is beginning the investigation with a fairly clear focus rather than a general focus, it is likely that interviews will be semi-structured so that they can address more specific issues.	Third-party data can evaluate the objective validity of participants' first-person accounts.	Aslan, I., Burkhardt, H., Kraus, J., & André, E. (2016); Zhu, B., Hedman, A., Feng, S., Li, H., & Osika, W. (2017); Salehzadeh Niksirat, K., Silpasuwanchai, C., Mohamed Hussien Ahmed, M., Cheng, P., & Ren, X. (2017); Prpa, M., Tatar, K., Riecke, B. E., & Pasquier, P. (2017); Buie, E. (2016); Belser, A. B., Agin-Liebes, G., Swift, T. C., Terrana, S., Devenot, N., Friedman, H. L., ... & Ross, S. (2017); Garcia-Romeu, A., Himelstein, S., & Kaminker, J. (2015)
**NEUROLOGICAL AND PHYSIOLOGICAL**								
Brain Lesions	Broca, P. (1861)	To show us how dynamic brain networks depend on the function of particular components, how cognition can be at least partially decomposed into modules of sorts, and how degeneracy and plasticity come into play.	Participants have portions of their brain removed. Comparison is made between performance before and after the lesion and consequent deficits are noted. One can also study participants whose brains have been damaged through natural means.	Usually done as a consequence of necessary survey (e.g., removing a tumour), so minimal consequence to the participant; Shows us insight into the causally necessary function of brain structures	Extremely invasive; Expensive; Low generalizability; Cannot be experimentally produced; Assumes that discrete anatomical modules deal with different cognitive functions.	When brain sugery is required, one can study its effects.	Test-retest is not applicable in these studies, so reliability is often not an interest; test-retest reliability that has been performed generates high coefficients. Neuroimaging data is used for validity tests.	Urgesi, C., Aglioti, S. M., Skrap, M., & Fabbro, F. (2010); Kastrup, B. (2017)
Electroencephalography (EEG)	Berger, H. (1929)	To show brain activity in certain psychological states.	Participants have electrodes placed on their face and scalp to directly measure brain electrical activity just under the scalp. Meditation types and their EEG brain waves: focused attention (beta 13-30Hz; gamma 30-50Hz), open monitoring (theta 4-8Hz), and automatic self-transcending (alpha-1 8-10Hz)	Consumer EEG headsets make this neuroimaging technique more accessable and affordable; Noninvasive; Good temporal resolution; Can be portable	Electric conductivity may vary widely from person to person and also over time, due to the natural conductivities of other tissues such as brain matter, blood, and bones. Because of this, it is sometimes unclear exactly which region of the brain is emitting a signal; Difficult to determine the source of the underlying signal; Gels can be messy; Clear signal usually requires a shaved head; Requires a reference	When you want to record real-time measures of brain activity.	EEG meets high standards of reliability and validity is demonstrated by hundreds of peer-reviewed journal articles – greater than 0.9 reliable. Content validity of qEEG is established by correlations with independent measures, e. g., MRI, PTE, SPECT.	Lehmann, D., Faber, P. L., Achermann, P., Jeanmonod, D., Gianotti, L. R., and Pizzagalli, D. (2001); Aftanas, L. I., and Golocheikine, S. A. (2002); Travis, F. & Arenander, A. (2004); Yamamoto, S., Kitamura, Y., Yamada, N., Nakashima, Y., & Kuroda, S. (2006); Kerr, C. E., Sacchet, M. D., Lazar, S. W., Moore, C. I., and Jones, S. R. (2013); Tenke, C. E., Kayser, J., Svob, C., Miller, L., Alvarenga, J. E., Abraham, K., ... & Bruder, G. E. (2017); Beauregard, M., Courtemanche, J., and Paquette, V. (2009); Amores, J., Benavides, X., & Maes, P. (2016); Choo, A., & May, A. (2014); Du Plessis, I. (2017); Gu, G., & Frasson, C. (2017); Hinterberger, T. (2011); Kosunen, I., Salminen, M., Järvelä, S., Ruonala, A., Ravaja, N., & Jacucci, G. (2016); Prpa, M., Cochrane, K., & Riecke, B. E. (2015); Das, N., & Gastaut, H. (1955); Banquet, J. P. (1973); Gervais, R., Roo, J. S., Frey, J., & Hachet, M. (2016); Antle, A. N., Chesick, L., & Mclaren, E.-S. (2018)

Name	Reference	Goal	Basic Methods	Advantages	Disadvantages	Suitability	Reliability and Validity	Who's using this method?
Magnetoencephalograph (MEG)	Cohen (1968)	To map brain activity by recording magnetic fields produced by electrical currents occurring naturally in the brain, using very sensitive magnetometers.	Participants may be positioned on a moveable examination table or seated in a comfortable chair within a room that shields out any electric and magnetic noise that could interfere with the exam. They will be positioned within the stationary helmet that contains the MEG detectors placed on the head.	Improved spatial resolution compared to EEG because magnetic fields are less distorted by bone (skull); Noninvasive; Activity is localized with more accuracy compared to EEG; Reference free; Direct measure of brain function	Not as good as fMRI at localizing activity; Specialized shielding is required to eliminate the magnetic interference; Requires highly sensitive instrumentation; Expensive; Not portable	Used for basic research into perceptual and cognitive brain processes, localizing regions affected by pathology before surgical removal, determining the function of various parts of the brain, and neurofeedback.	Reliability and validity reports of MEG are strong, yet methodological questions regarding optimal modeling techniques remain.	Yamada, N., Nakashima, Y., & Kuroda, S. (2006); Kerr, C. E., Sacchet, M. D., Lazar, S. W., Moore, C. I., and Jones, S. R. (2013); Dor-Ziderman, Y., Berkovich-Ohana, A., Glicksohn, J., & Goldstein, A. (2013); Berkovich-Ohana, A., Dor-Ziderman, Y., Glicksohn, J., & Goldstein, A. (2013); Wong, W. P., Camfield, D. A., Woods, W., Sarris, J., & Pipingas, A. (2015)
Positron Emission Tomography (PET)	Phelps, M. E., Hoffman, E. J., Mullani, N. A., & Ter-Pogossian, M. M. (1975)	To illustrate where neural firing is taking place.	Participants have a small amount of radioactive tracer injected into a vein. The tracer enters the brain after approximately 30s, and in the following 30s radiation in the brain rises to its maximal value; a picture of the regional Cerebral Blood Flow is taken during this time frame.	Researchers can choose which tracer to synthesize, e.g., serotonin or glutamate; Little artifacts; High spatial resolution; Quick scan times	We can only locate generalized areas of brain activity and not specific locations; Expensive; Invasive; Not suitable for children or vulnerable populations.	Important for understanding the role of various neurotransmitters in cognition.	Test–retest reliability, except for language mapping, is good, with variance ranging from 2-12%. Good validity	Kjaer, T. W., Bertelsen, C., Piccini, P., Brooks, D., Alving, J., & Lou, H. C. (2002); Borg, J., Andrée, B., Soderstrom, H., & Farde, L. (2003); Hakamata, Y., Iwase, M., Kato, T., Senda, K., & Inada, T. (2013); Karlsson, H., Hirvonen, J., Salminen, J. K., & Hietala, J. (2011); Herzog, H., Lele, V. R., Kuwert, T., Langen, K. J., Kops, E. R., & Feinendegen, L. E. (1990); Lou, H. C., Kjaer, T. W., Friberg, L., Wildschiodtz, G., Holm, S., & Nowak, M. (1999); Vollenweider, F. X., Leenders, K. L., Scharfetter, C., Maguire, P., Stadelmann, O., and Angst, J. (1997); Gouzoulis-Mayfrank, E., Schreckenberger, M., Sabri, O., Arning, C., Thelen, B., Spitzer, M., ... & Sass, H. (1999)
Single Photon Emission Computed Tomography (SPECT)	Kuhl, D. E., & Edwards, R. Q. (1963)	To render a 3D map of a person's body (including brain).	Participants are administered a radioactive compound and observed by a "gamma camera," which is sensitive to gamma radiation.	Permits accurate localization in 3D space; Widely available; Relatively inexpensive compared to PET; Longer imaging time compared to PET;	Long scan times; Low-resolution images prone to artifacts and attenuation; Still fairly costly	SPECT can be used to complement any gamma imaging study, where a true 3D representation can be helpful, e.g., tumor imaging, infection (leukocyte) imaging, thyroid imaging or bone scan. It can be used to provide information about localized function in internal organs, such as functional cardiac or brain imaging.	There are few consistent data regarding validity and realiability for SPECT. There is a high rate of false positives.	Newberg, A., Alavi, A., Baime, M., Pourdehnad, M., Santanna, J., & d'Aquili, E. (2001); d'Aquili, E. G., & Newberg, A. B. (1993); Riba, J., Romero, S., Grasa, E., Mena, E., Carrió, I., & Barbanoj, M. J. (2006); Hermle, L., Gouzoulis-Mayfrank, E., & Spitzer, M. (1998); Sanches, R. F., de Lima Osório, F., dos Santos, R. G., Macedo, L. R., Maia-de-Oliveira, J. P., Wichert-Ana, L., ... & Hallak, J. E. (2016)
Magnetic Resonance Imaging (MRI) and fMRI	Lauterbur, P. C. (1973)	To measure brain structure and activity.	Participants are subjected to a powerful magnet that aligns the hydrogen nuclei of water atoms inside of their brain and can determine changes in the amount of brain tissue or in the amount of blood flow. Typically, the more active a particular brain structure, the more blood flow it receives. MRI has also been used to quantify the volume of particular brain structures.	Non-invasive; pose little health risk; can be used in all ages, including in-utero; wide spread availability; relatively low cost per scan; good spatial resolution; better temporal resolution than other indirect neuroimaging methods, although not as good as EEG.	Expensive; Not portable; Poor temporal resolution; Very noisy; Cannot be used to evaluate neurotransmitter system;	When you want to measure short term and long term changes in the brain; fMRI can be used to produce activation maps showing which parts of the brain are involved in a particular mental process.	There is no agreed-upon average value for fMRI reliability. There are so many factors spread out across so many levels of influence that it is almost impossible to summarize the reliability of fMRI with a single value. Most fMRI results are reliable in the intra-class correlation (ICC) = 0.33 to 0.66 range.	Lazar, S. W., Bush, G., Gollub, R. L., Fricchione, G. L., Khalsa, G., & Benson, H. (2000); Johnstone, B., Cohen, D., Bryant, K. R., Glass, B., & Christ, S. E. (2015); Wang, D. J. J., Rao, H., Korczykowski, M., Wintering, N., Pluta, J., Khalsa, D. S., et al. (2011); Vago, D. R., and Silbersweig, D. A. (2012); Miller, L., Balodis, I. M., McClintock, C. H., Xu, J., Lacadie, C. M., Sinha, R., & Potenza, M. N. (2018); Beauregard, M., Courtemanche, J., and Paquette, V. (2009); Immordino-Yang, M. H., McColl, A., Damasio, H., & Damasio, A. (2009); van Elk, M., Arciniegas Gomez, M. A., van der Zwaag, W., van Schie, H. T., & Sauter, D. (2019); Daumann, J., Wagner, D., Heekeren, K., Neukirch, A., Thiel, C. M., & Gouzoulis-Mayfrank, E. (2010); Carhart-Harris, R. L., Erritzoe, D., Williams, T., Stone, J. M., Reed, L. J., Colasanti, A., ... & Hobden, P. (2012); Lebedev, A. V., Lövdén, M., Rosenthal, G., Feilding, A., Nutt, D. J., & Carhart-Harris, R. L. (2015); Carhart-Harris, R. L., Muthukumaraswamy, S., Roseman, L., Kaelen, M., Droog, W., Murphy, K., ... & Leech, R. (2016); Tagliazucchi, E., Roseman, L., Kaelen, M., Orban, C., Muthukumaraswamy, S. D., Murphy, K., ... & Bullmore, E. (2016); Palhano-Fontes, F., Andrade, K. C., Tofoli, L. F., Santos, A. C., Crippa, J. A. S., Hallak, J. E., ... & de Araujo, D. B. (2015); de Araujo, D. B., Ribeiro, S., Cecchi, G. A., Carvalho, F. M., Sanchez, T. A., Pinto, J. P., ... & Santos, A. C. (2012); Travis, F., Nash, J., Parim, N., & Cohen, B. H. (2020); Ritskes, R., Ritskes-Hoitinga, M., Stodkilde-Jorgensen, H., Baerentsen, K., and Hartman, T. (2003)
Near-Infrared Spectroscopy (NIRS) and fNIRS	Jöbsis (1977)	To measure the relative blood flow (oxygenation level) from brain activation.	Participants wear a cap or headband with electrodes on them that measure scalp signals.	Non-invasice, low-cost, safe, portable, can be used with other imaging techniques, high temporal resolution, less prone to movement artifacts	Low spatial resolution, brain sensitivity, and penetration depth	When you want a small, wearable device, especially good for neuromonitoring and neurorehabilitation. Good when movement is a factor.	fNIRS may be a reliable tool for emotion studies, especially for a group analysis and under larger spatial scales. However, the individual-level and the channelwise fNIRS prefrontal responses were not sufficiently stable.	Hu, X., Zhuang, C., Wang, F., Liu, Y. J., Im, C. H., & Zhang, D. (2019); Gundel, F., von Spee, J., Schneider, S., Haeussinger, F. B., Hautzinger, M., Erb, M., ... & Ehlis, A. C. (2018); Zheng, Y. L., Wang, D. X., Zhang, Y. R., & Tang, Y. Y. (2019); Scholkmann, F., Holper, L., Preller, K. H., & Vollenweider, F. X. (2019)
GooseCam – philoerection	Benedek, M., Wilfling, B., Lukas-Wolfbauer, R., Katzur, B. H., & Kaernbach, C. (2010)	To investigate goosebumps as a response to strong emotional experiences.	Participants wear an optical recording device that enables video recordings from the skin surface. Data are then analysed by means of spectral analysis (2D discrete Fourier transformation).	Non-invasive; Increased objectivity; Continuous measure; Robust to variations in skin characteristics	Positive emotions can occur without philoerection;	Objective measure of "chills" or shivers produced from emotional experiences such as awe.	Reduced internal validity because this method does not distinguish between the subjective sensation of chills and the objective phenomenon of visible piloerection.	Uchida, M., Akaho, R., Ogawa, K., & Tsumura, N. (2018); Sumpf, M., Jentschke, S., & Koelsch, S. (2015); Wassiliwizky, E., Jacobsen, T., Heinrich, J., Schneiderbauer, M., & Menninghaus, W. (2017); Quesnel, D., & Riecke, B. E. (2017); Benedek, M., & Kaernbach, C. (2011); Quesnel, D., Stepanova, E. R., Aguilar, I., Pennefather, P., and Riecke, B. E. (2018); Stepanova, E. R., Quesnel, D., & Riecke, B. E. (2019)
Pneumograph – respiration	Marey, E. J. (1878)	To record velocity and force of chest movements during respiration.	Participants wear one or more straps around their chest and abdomen, which measures the expansion and contraction (i.e., volume) of their breathing.	Noninvasive; Inexpensive; Easy to Use; Does not require extensive training; Allows participant to see breathing cycle	Indirect measure; Sensitive to motion; May feel restrictive	Can help evaluate respiratory mechanism; Can be used for biofeedback; Often used in polygraph tests because increased rate and volume of respiration = stress; Should be used in conjunction with other measures	Internal validity threat is the Hawthorne effect: people behave differently when they know that they are being observed; External validity threat is using convenience sample, which is not representative enoug of the population; Huge range in individual differences and indirect measures of self-transcendence makes reliability challenging	Benedek, M., & Kaernbach, C. (2011); Mori, K., & Iwanaga, M. (2017); Shiota, M. N., Neufeld, S. L., Yeung, W. H., Moser, S. E., & Perea, E. F. (2011); Davies, C., & Harrison, J. (1996); Du Plessis, I. (2017); Hinterberger, T. (2011); Vidyarthi, J., & Riecke, B. E. (2013); Kitson, A., Riecke, B. E., & Vidyarthi, J. (2014); Prpa, M., Cochrane, K., & Riecke, B. E. (2015); Prpa, M., Tatar, K., Riecke, B. E., & Pasquier, P. (2017); Roo, J. S., Gervais, R., & Hachet, M. (2016); Van Rooij, M., Lobel, A., Harris, O., Smit, N., & Granic, I. (2016); Shaw, C. D., Gromala, D., & Seay, A. F. (2007); Banquet, J. P. (1973); Gervais, R., Roo, J. S., Frey, J., & Hachet, M. (2016)

Name	Reference	Goal	Basic Methods	Advantages	Disadvantages	Suitability	Reliability and Validity	Who's using this method?
Electrodemograph (EDG) – skin electrical activity	DuBois-Reymond (1849)/Vigouroux, R. (1879)	To measure skin electrical activity directly (skin conductance and skin potential) and indirectly (skin resistance) using electrodes placed over the digits or hand and wrist.	Participants wear electrodes on the palm of the hand or sole of the foot. The skin acts like a series of electrical resistors wired in parallel. As sweat increases in the ducts, the resistance at the skin goes down, resulting in higher levels of recorded skin conductance.	Easy to use; Noninvasive; Relatively inexpensive; Skin conductance is a reliable measure of emotional arousal – reflects the level of activation within the appetitive and aversive emotional/motivational systems.	Measures only emotional arousal, not valence.	Psychological states that reliably evoke changes in skin conductance are tied to stimuli of pictures, music, computer game avatars, and emotional films. GSR is a possible indicator of the orienting response (OR), reflective of automatic increase in attention paid to novel or signal stimuli. Skin conductance could, however, reflect the emotional-motivational component of the OR due to sympathetic nervous system activity. There is a strong, reliable association between skin conductance and sympatetic nervous system. Electrodermal activity is a reliable indicator of stress because it innervates the peripheral nervous system only and is easy to measure on the hand.	Internal validity threat is the Hawthorne effect: people behave differently when they know that they are being observed; External validity threat is using convenience sample, which is not representative enoug of the population; Huge range in individual differences and indirect measures of self-transcendence makes reliability challenging	Benedek, M., & Kaernbach, C. (2011); Mori, K., & Iwanaga, M. (2017); Shiota, M. N., Neufeld, S. L., Yeung, W. H., Moser, S. E., & Perea, E. F. (2011); Bernal, G., & Maes, P. (2017); Du Plessis, I. (2017); Gromala, D., Tong, X., Choo, A., Karamnejad, M., & Shaw, C. D. (2015); Hinterberger, T. (2011); Shaw, C. D., Gromala, D., & Seay, A. F. (2007); Gervais, R., Roo, J. S., Frey, J., & Hachet, M. (2016)
Photoplethysmograph (PPG) – blood flow changes	Hertzman, A. B. (1937)	To measure the relative blood flow through a digit using a photoplethysmographic (PPG) sensor attached by a Velcro band to the fingers or to the temple to monitor the temporal artery.	Participants wear a sensor that wraps around or clips onto their finger. A signal is sent either through the finger or is reflected back by tissue. Less light is absorbed when blood flow is greater, increasing the intensity of light reaching the sensor.	Can measure blood volume pulse (BVP), heart rate, and heart rate variability (HRV); More sensitive than a thermistor to minute blood flow changes; Simple to use; Inexpensive	Indirect measure of heart rate; Motion artefacts; Need for a potentiometer to adjust the signal	Biofeedback therapists can use a photoplethysmograph to supplement temperature biofeedback when treating chronic pain, edema, headache (migraine and tension-type headache), essential hypertension, Raynaud's disease, anxiety, and stress.	PPG signal is strong and robust; Internal validity threat is the Hawthorne effect: people behave differently when they know that they are being observed; External validity threat is using convenience sample, which is not representative enoug of the population; Huge range in individual differences and indirect measures of self-transcendence makes reliability challenging	Benedek, M., & Kaernbach, C. (2011); Bernal, G., & Maes, P. (2017); Chittaro, L., Sioni, R., Crescentini, C., & Fabbro, F. (2017); Du Plessis, I. (2017); Hinterberger, T. (2011); Roo, J. S., Gervais, R., & Hachet, M. (2016); Shaw, C. D., Gromala, D., & Seay, A. F. (2007)
Electroggardiograph (ECG/EKG) – heart rate	Einthoven, W. (1895)	To measure the electrical activity of the heart by using electrodes placed on the skin.	Participants wear electrodes placed on the torso, wrists, or legs, to measure the electrical activity of the heart and measures the interbeat interval (distances between successive R-wave peaks in the QRS complex). The interbeat interval, divided into 60 seconds, determines the heart rate at that moment. The statistical variability of that interbeat interval is what we call heart rate variability.	ECG method is more accurate than the PPG method in measuring heart rate variability; Safe; Inexpensive; Easy to Use; Widely available	Heart activity is innervated by both the CNS and PNS, so it can be difficult to determine the nature of "stress", i.e., whether it's positive or negative; One must account for baselines and individual difference when comparing HRV; Restricts body movement	Biofeedback therapists use heart rate variability (HRV) biofeedback when treating asthma, COPD, depression, anxiety, fibromyalgia, heart disease, and unexplained abdominal pain. Research shows that HRV biofeedback can also be used to improve physiological and psychological wellbeing in healthy individuals.	Internal validity threat is the Hawthorne effect: people behave differently when they know that they are being observed; External validity threat is using convenience sample, which is not representative enoug of the population; Huge range in individual differences and indirect measures of self-transcendence makes reliability challenging	Sumpf, M., Jentschke, S., & Koelsch, S. (2015); Mori, K., & Iwanaga, M. (2017); Shiota, M. N., Neufeld, S. L., Yeung, W. H., Moser, S. E., & Perea, E. F. (2011); Das, N., & Gastaut, H. (1955); Gervais, R., Roo, J. S., Frey, J., & Hachet, M. (2016)
Electromyograph (EMG) – muscle electrical activity	Hardyck, C. D., Petrinovich, L. F., & Ellsworth, D. W. (1966)	To measure the electrical signal associated with muscle activity	Participants wear one or more active electrodes that are placed over a target muscle and a reference electrode that is placed within six inches of either active.	Can be a useful diagnostic tool; Surface EMG easy and quick to apply; Minimal discomfort	Adipose tissue (fat) can affect EMG recordings; Compliant skin needed for accrate reading; Muscle cross talk can occur; Surface EMG is limited due to lack of deep muscles reliability; No standard placement	Specific facial muscles involved in the valence of emotional processessing during media use: corrugator supercilii, orbicularis oculi, and zygomaticus major. If interested in discrete emotions one should pair facial EMG with existing reliable and valid self-report scales	Internal validity threat is the Hawthorne effect: people behave differently when they know that they are being observed; External validity threat is using convenience sample, which is not representative enoug of the population; Huge range in individual differences and indirect measures of self-transcendence makes reliability challenging	Austin, J. H. (2010); Das, N., & Gastaut, H. (1955); Banquet, J. P. (1973)
**BEHAVIOURAL**								
Observation	-	To watch (i.e. observe) behavior as it organically and spontaneously unfolds in a natural environment.	Participants are either aware (overt) or not (covert) they are being studied. Observations can be in a controlled setting, in a natural environment, or the researcher can join in as part of the group. Behaviours can be coded	Access to people in real life situations; Can be used when questionnaires or interviewing is inappropriate or not possible; Explains meaning and context; Inexpensive	Can be viewed as too subjective; Time consuming; Depends on researcher role; Overt may affect validity of findings; Covert may be unethical; high potential for role conflict; May not tell the whole story	Observational research is used when other data collection procedures, such as surveys, questionnaires, etc. are not effective or adequate. Also used when the goal is to evaluate an ongoing behaviour process, event, or situation; or when there are physical outcomes that can be readily seen.	Strong on validity and in-depth understanding; Researchers can become too involved and thus reduce vaildity; Natural observations are less reliable; Hawthorne effect can affect validity	Quesnel, D., Stepanova, E. R., Aguilar, I., Pennefather, P., and Riecke, B. E. (2018); Gervais, R., Roo, J. S., Frey, J., & Hachet, M. (2016); Van Rooij, M., Lobel, A., Harris, O., Smit, N., & Granic, I. (2016); Antle, A. N., Chesick, L., & Mclaren, E. -S. (2018); Uthaug, M. V., Van Oorsouw, K., Kuypers, K. P. C., Van Boxtel, M., Broers, N. J., Mason, N. L., ... & Ramaekers, J. G. (2018); Coward, D. D. (1990)
Body size estimation	van Elk, M., Karinen, A., Specker, E., Stamkou, E., & Baas, M. (2016)	To measure effects of awe on the perception of one's body.	Participants were presented with a graphical representation of the main entrance of the church and were required to indicate which figure best represented the perceived size of their body. Participants who report stronger feelings of awe tend to perceive their body as smaller.	Easy to use; low-cost; graphical representation	Does not account for body schema, the implicit perception of one's body that underlies our actions in the world.	Measures the conscious and subjective perception of one's body, which has been referred to in the literature as 'body image'.	No strong validation of measure at this time.	-
Zimbardo Time Perspective Inventory (ZTPI-F)	Zimbardo et al. (1997); Zimbardo and Boyd (1999)	To measure effects of awe on perceived time availability.	Participants rate their agreement with each statement on a 5-point Likert scale ranging from 1 (strongly disagree) to 5 (strongly agree). 13 questions for future factors as a future time perspective questionnaire.	Easy to use; low-cost; short	Mostly validated in adolescent and early adult populations only. A recent study showed experimentally induced awe does not affect time perception.	Measure of time perception as a correlate of awe.	Cronbach's α for this scale was 0.78	Rudd, M., Vohs, K. D., and Aaker, J. (2012)
Small Self	Piff, P. K., Dietze, P., Feinberg, M., Stancato, D. M., & Keltner, D. (2015)	To measure effects of awe on the perception of a dimished sense of self.	Participants are given a page of 23x40 cubic graph paper that includes grass at the bottom of the page and a sun at the top-right corner. They are asked to draw themselves and they can add other people or objects. When they finish drawing, they write a "Me" next to their self image. To measure perceived self-size, record the number of squares included within the figure of the participant's drawn self. This number includes any squares the pencil line touched, and blank squares included in the body of the figure.	Easy to use; low-cost; short	Some people are self-conscious of their drawing skills; low reliability	Awe intensity is significantly correlated with the composite index of perceived self-size.	Cronbach's └alpha┐ .51	Bai, K., Maruskin, L. A., Chen, S., Gordon, A. M., Stellar, J. E., McNeil, G. D., ... & Keltner, D. (2017)
Prosocial Tendencies Measure (PTM)	Carlo and Randall (2002)	To assess six dimensions of prosocial behaviour: public, anonymous, dire, emotional, compliant, and altruism.	Participants are asked to rate the extent to which the 26 statements described themselves on a 5-point scale ranging from 1 (Does not describe me at all) to 5 (Describes me well).	Easy to use; low-cost; short	Mostly validated in adolescent and early adult populations only.	Measures pro-social behaviour outcomes from self-transcendence in adolescents	Cronbach's alpha for this scale was 0.92. Convergent: PTM-R scales were significantly related with theoretically relevant variables (e. g., sympathy, perspective taking, moral reasoning) and non significantly related with non theoretically relevant variables (e.g., vocabulary skills, social desirability, personal distress)	Kou et al. (2007); Azimpour, A., Neasi, A., Shehni-Yailagh, M., & Arshadi, N. (2012); Carlo, G., Hausmann, A., Christiansen, S., & Randall, B. A. (2003); Vinothkumar, M. (2015); Pandya, S. P. (2017)
